# Targeted screening of inflammatory mediators in spontaneous degenerative disc disease in dogs reveals an upregulation of the tumor necrosis superfamily

**DOI:** 10.1002/jsp2.1292

**Published:** 2023-11-23

**Authors:** Thomas Bitterli, David Schmid, Ladina Ettinger, Olga Krupkova, Frances C. Bach, Marianna A. Tryfonidou, Björn P. Meij, Antonio Pozzi, Frank Steffen, Karin Wuertz‐Kozak, Lucas A. Smolders

**Affiliations:** ^1^ Clinic for Small Animal Surgery, Department for Small Animals, Vetsuisse Faculty University of Zurich Zurich Switzerland; ^2^ Spine Surgery University Hospital Basel Basel Switzerland; ^3^ Department of Biomedicine University of Basel & University Hospital Basel, Tissue Engineering Basel Switzerland; ^4^ Department of Clinical Sciences, Faculty of Veterinary Medicine Utrecht University Utrecht the Netherlands; ^5^ Institute for Biomechanics, ETH Zurich Zurich Switzerland; ^6^ Department of Biomedical Engineering Rochester Institute of Technology (RIT) Rochester New York USA; ^7^ Schön Clinic Munich Harlaching, Spine Center Academic Teaching Hospital and Spine Research Institute of the Paracelsus Medical University Salzburg (Austria) Munich Germany

**Keywords:** back pain, degenerative disc disease, dog, inflammation, nerve growth factor, RANKL, tumor necrosis factor superfamily

## Abstract

**Background:**

The regulation of inflammatory mediators in the degenerating intervertebral disc (IVD) and corresponding ligamentum flavum (LF) is a topic of emerging interest. The study aimed to investigate the expression of a broad array of inflammatory mediators in the degenerated LF and IVD using a dog model of spontaneous degenerative disc disease (DDD) to determine potential treatment targets.

**Methods:**

LF and IVD tissues were collected from 22 normal dogs (Pfirrmann grades I and II) and 18 dogs affected by DDD (Pfirrmann grades III and IV). A qPCR gene array was used to investigate the expression of 80 inflammatory genes for LF and IVD tissues, whereafter targets of interest were investigated in additional tissue samples using qPCR, western blot (WB), and immunohistochemistry.

**Results:**

Tumor necrosis factor superfamily (TNFSF) signaling was identified as a regulated pathway in DDD, based on the significant regulation (*n*‐fold ± SD) of various TNFSF members in the degenerated IVD, including *nerve growth factor* (*NGF*; −8 ± 10), *CD40LG* (464 ± 442), *CD70* (341 ± 336), *TNFSF Ligand 10* (9 ± 8), and *RANKL/TNFSF Ligand 11* (85 ± 74). In contrast, TNFSF genes were not significantly affected in the degenerated LF compared to the control LF. Protein expression of NGF (WB) was significantly upregulated in both the degenerated LF (4.4 ± 0.5) and IVD (11.3 ± 5.6) compared to the control group. RANKL immunopositivity was significantly upregulated in advanced stages of degeneration (Thompson grades IV and V) in the nucleus pulposus and annulus fibrosus of the IVD, but not in the LF.

**Conclusions:**

DDD involves a significant upregulation of various TNFSF members, with tissue‐specific expression profiles in LF and IVD tissues. The differential involvement of TNFSF members within multiple spinal tissues from the same individual provides new insights into the inflammatory processes involved in DDD and may provide a basis to formulate hypotheses for the determination of potential treatment targets.

## Graphical abstract

The study aimed to investigate the expression of inflammatory mediators in the degenerated ligamentum flavum (LF) and intervertebral disc (IVD) and to identify key inflammatory targets using a dog model of spontaneous degenerative disc disease (DDD). DDD involves a significant upregulation of various tumor necrosis factor superfamily (TNFSF) members, with tissue‐specific expression profiles in LF and IVD tissues. The differential involvement of TNFSF members within multiple spinal tissues from the same individual provides new insights into the inflammatory processes involved in DDD and may provide a basis to formulate hypotheses for the determination of potential treatment targets.

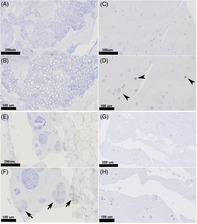

## 1 INTRODUCTION

Back pain is a common health issue with considerable socio‐economic consequences.^1^ Degenerative disc disease (DDD) is one of the major causes for back pain, involving degenerative changes of different spinal structures, including the intervertebral disc (IVD) and spinal ligaments. Degeneration of these structures can lead to pain originating from compression of spinal nerves or from nerves located within these degenerating structures.^2^

Besides people, the dog is the only animal species known to frequently suffer from spontaneous DDD and back pain.^3^ In particular, the syndrome called degenerative lumbosacral stenosis observed in medium‐ to large‐breed dogs resembles chronic low back pain in people, involving degeneration of the IVD and other spinal structures.^4^, ^5^ Because of the significant resemblance in the characteristics of DDD, the dog has frequently been used as an animal model for studying spontaneous (i.e., not experimentally induced) DDD.^6^, ^7^, ^8^

Apart from the IVD, the ligamentum flavum (LF), which is located in between two adjacent vertebrae posterior/dorsal to the spinal cord, is known to be involved in degenerative spinal disease in humans and dogs.^9^, ^10^ Degeneration of the LF in people involves fibrosis and ossification of the ligament.^9^, ^11^ Through these mechanisms, the LF is considered a causative factor in low back pain, mainly through compression or stimulation of neural structures surrounding the LF.^9^

Although DDD can be a major cause for pain, degeneration of spinal ligaments and the IVD is also a common finding in asymptomatic individuals.^12^, ^13^ More recent evidence suggests that pro‐inflammatory mediators within degenerating tissues are key molecules that induce pain in degenerating spinal structures.^14^, ^15^ In addition, pro‐inflammatory mediators within degenerating spinal structures are believed to contribute to tissue catabolism, resulting in further deterioration of tissue structure and function.^16^, ^17^ Therefore, research aimed at identifying inflammatory mediators involved in spinal degeneration and pain has received considerable attention in both veterinary and human medicine over the past decade.^15^, ^18^

Currently, there are still gaps in the scientific literature on the inflammatory processes linked to chronic low back pain. First, the contribution of neighboring spinal ligaments to back pain has been largely ignored. Second, when reviewing the approaches used to investigate inflammatory and pain‐related factors, the majority of studies^15^, ^18^ analyze only a small group of pre‐selected targets (e.g., *TNF‐α* and *IL‐1β*) instead of investigating a wide array of other, potentially also relevant biomolecular factors. Thirdly, the majority of the reported findings on inflammatory mediators and pain have been gathered in vitro and studies using in vivo material have mainly used animals in which degeneration was not spontaneous, but induced.^15^

Therefore, the aim of this study was to investigate the expression of a broad array of inflammatory mediators in the degenerated LF and IVD using a dog model of spontaneous DDD, with the aim to determine potential treatment targets for DDD.

## 2 MATERIALS AND METHODS

### 2.1 Animals

Tissues were collected from 22 dogs free of spinal pathology (control group) and 18 dogs with degenerative lumbosacral stenosis (DDD group; Table 1). The control group included dogs that were euthanized due to reasons unrelated to DDD at the Clinic for Small Animal Surgery, Vetsuisse Faculty Zurich (client‐owned dogs; tissues were collected only after obtaining owner's consent; Statement from Swiss Ethical committee: Appendix I) or at the Department of Clinical Sciences, Utrecht University, the Netherlands (experimental animals; experiment approved by the Ethical Committee of Utrecht University, DEC number 2013.III.08.054).

**TABLE 1:** Overview of samples (breed, age, gender, and tissue collected) of the control and DDD group used for Reference gene analysis (RGA), qPCR array analysis (Array), qPCR of tumor necrosis factor superfamily target genes (qPCR), and western blot (WB) analysis. The Pfirrmann Grade (PG), severity of disc protrusion, and type of Modic Changes (MC) have been listed for each dog.

Nr.	Breed	Age	Gender	Tissue	PG	Protrusion	MC	Use
**Control group**								
1	Beagle	2y 11m	F	LF, IVD	II	No	No	RGA
2	German Shepherd Dog	10y 11m	MC	LF, IVD	II	Mild	No	RGA
3	Beagle	2y 8m	F	LF, IVD	I	No	No	RGA, WB
4	Bernese Mountain Dog	6Y	M	LF	II	Mild	No	RGA
5	Great Dane	5y 7m	M	LF	II	Mild	No	RGA
6	Mixed breed	13y	FC	LF	II	Mild	Type 3	RGA
7	Malinois	7y 6m	M	LF, IVD	II	Mild	No	RGA, WB
8	St. Bernard	2y 10m	M	LF	I	No	No	RGA
9	Beagle	2y 1m	F	IVD	II	No	No	RGA
10	Beagle	3y 2m	F	LF, IVD	II	No	No	Array, qPCR, WB
11	Beagle	3y 2m	F	LF, IVD	II	No	No	Array, qPCR
12	Greater Swiss Mountain Dog	6y 1m	F	LF, IVD	II	No	No	Array, qPCR
13	Beagle	1y 10m	F	LF	I	No	No	Array
14	Beagle	2y 2m	F	LF, IVD	II	No	No	Array, qPCR, WB
15	Beagle	1y 10m	F	LF	II	No	No	qPCR, WB
16	Beagle	1y 8m	F	LF	I	No	No	qPCR
17	Mixed Breed	4y 9m	FC	LF	I	No	No	qPCR
18	Beagle	1y 8m	F	IVD	II	No	No	qPCR
19	Beagle	1y 10m	F	IVD	II	No	No	qPCR, WB
20	Beagle	2y 1m	F	IVD	I	No	No	qPCR
21	Labrador Retriever	3y 4m	MC	IVD	II	Mild	No	qPCR, WB
22	German Shepherd Dog	4y 6m	MC	LF, IVD	II	Mild	No	qPCR
**DDD group**								
1	German Shepherd Dog	9y 5m	MC	LF, IVD	III	Severe	No	RGA
2	German Shepherd Dog	5y 9m	M	LF	III	Severe	Type 3	RGA
3	Newfoundlander	3y 3m	FC	LF	III	Severe	No	RGA
4	Labrador Retriever	11y 9m	M	LF	IV	Severe	Type 3	RGA
5	German Shepherd Dog	3y	M	LF	III	Moderate	Type 3	RGA
6	German Shepherd Dog	8y 11m	MC	LF, IVD	IV	Severe	Mixed 1 + 3	RGA, Array
7	Continental bulldog	7y 3m	MC	IVD	III	Moderate	No	RGA
8	Bernese Mountain Dog	8y 8m	F	IVD	III	Severe	No	RGA
9	Leonberger	2y 4m	M	LF, IVD	IV	Severe	Type 3	Array, qPCR, WB
10	Mixed Breed	9y	M	LF, IVD	III	Moderate	Type 2	Array, qPCR, WB
11	German Shepherd Dog	5y 9m	M	LF	III	Severe	Type 3	Array
12	German Shepherd Dog	8y 11m	MC	LF	III	Severe	Type 3	Array
13	Berger Blanc Suisse	5y 10m	M	LF, IVD	III	Moderate	No	Array, qPCR, WB
14	German Shepherd Dog	5y 6m	M	LF, IVD	III	Severe	Type 3	Array, qPCR, WB
15	German Shepherd Dog	3y	M	LF, IVD	III	Moderate	Type 3	qPCR
16	German Pointer	12y	F	LF, IVD	IV	Severe	Type 3	qPCR
17	Labrador retriever	11y 4m	MC	IVD	IV	Severe	No	qPCR
18	Mixed Breed	12y 7m	MC	LF	III	Moderate	No	qPCR

The DDD group included client‐owned dogs diagnosed with low back pain due to degenerative lumbosacral stenosis (collected both at the University of Zurich and Utrecht University). Informed owner consent for using LF and IVD tissues for scientific use was obtained for all animals. The diagnosis of back pain originating from degenerative lumbosacral stenosis was made by way of clinical examination performed by a board‐certified veterinary surgeon or neurologist (involving subjective gait analysis in walk and trot, palpation and mobility assessment of the lumbosacral spine, and examination of conscious proprioception and reflexes of the pelvic limbs) and magnetic resonance imaging (MRI) of the lumbosacral spine.^19^

### 2.2 Assessment of degenerative state

To assess the degenerative state of the LF and IVD, MRI of the lumbosacral spine of all dogs was performed (MRI; either 3.0 T Philips Ingenia, Philips healthcare, or 1.5 T Magnetom open Viva, Siemens AG). The LF and IVD of the lumbosacral junction were assessed for the following:

1. Thickening/hypertrophy of the LF (absent or present).^10^
2. Degeneration of the IVD according to Pfirrmann (Stages I [healthy]–V [completely degenerated]).^20^, ^21^
3. Protrusion of the lumbosacral IVD (absent or present) with secondary compression of the cauda equina nerve roots.

### 2.3 Tissue sample collection

After diagnosis, dogs affected by degenerative lumbosacral stenosis were treated by standard‐of‐care surgery.^19^ Surgery consisted of dorsal laminectomy and discectomy, involving removal of the dorsal vertebral lamina of L7 and S1, en block removal of the LF, and partial removal of the IVD (partial annulectomy and nucleus pulpectomy). As such, tissue collection was an inherent part of the standard‐of‐care treatment of these patients. The nucleus pulposus and annulus fibrosus (AF) of the IVD were collected as a single piece of tissue as distinguishing these tissues in the degenerated, surgically collected samples proved challenging. For standardization purposes, LF and IVD tissues of the control group dogs were collected through the same approach. After removal, the samples of LF and IVD tissues were split, creating 2 complementary parts of equal size, snap‐frozen in liquid nitrogen, and subsequently stored at −80°C. Samples were used for different experimental purposes (i.e., qPCR gene array analysis, qPCR analysis of additional gene targets, and western blot [WB] analysis; see below and Table 1).

### 2.4 Classification of tissue samples

Tissue samples were assigned to either the control group or DDD group based on clinical examination and MRI of the collected tissues.

Samples for the control group were collected from normal dogs with no clinical signs of DDD. LF tissue samples were included if thickening/hypertrophy of the LF was absent on MRI. IVD samples were assigned to the control group if the L7‐S1 IVD had a Pfirrmann score of I and II and showed no to mild dorsal IVD protrusion (0%–25% of vertebral canal stenosis).^22^

Samples for the DDD group were collected from the dogs clinically diagnosed with DLSS. LF tissue samples were included in the DDD group if LF thickening/hypertrophy was evident on imaging. IVD samples were assigned to the DDD group if the L7‐S1 IVD had a Pfirrmann score of III and IV and showed moderate (25%–50% of vertebral canal stenosis) to severe (>50% of vertebral canal stenosis) dorsal IVD protrusion on imaging.^22^

The Pfirrmann Grade, degree of protrusion of the L7‐S1 IVD, and the presence and type of Modic changes (MCs) of the L7‐S1 vertebral endplates were recorded for each dog (Table 1). If possible, both LF and IVD tissues from the same animal were used for multiple experimental uses.

### 2.5 Identification of key inflammatory pathways

#### 2.5.1 qPCR gene array

Four (4) LF and IVD samples from both the control and DDD groups were used for qPCR array analysis (samples were selected based on RNA integrity [RIN] value; see below). Total RNA was isolated using the Nucleospin® RNA kit (Marchery‐Nagel, Oensingen, Switzerland) as instructed by the manufacturer (full description of the RNA isolation protocol: Supporting File 1). RNA was quantified spectrophotometrically using Nanodrop ND‐1000 (Nanodrop, Thermo Fisher Scientific) and RIN numbers were determined using a Bioanalyzer 2100 (Agilent Technologies). For the LF, RNA yields ranged from 76.1 to 321.8 ng/μL and 51.3 to 207.8 ng/μL for the control and DDD groups, respectively; for the IVD, RNA yields ranged from 21.8 to 378 ng/μL and 9.7 to 175 ng/μL for the control and DDD samples, respectively (RNA volume per sample: 40uL). RIN values of the samples ranged from 4.5 to 9.7 for the LF and 3.8 to 6.3 for the IVD, indicating that these samples could be used to perform valid gene expression analysis.^23^ cDNA was created using iScript™ cDNA Synthesis Kit and Bio‐Rad T100 Thermal Cycler (Bio‐Rad), using a total RNA amount of 1000 ng per sample (RNA amount needed to perform the array according to the manufacturer's instructions). Because of the relatively small RNA yield of the IVD samples, 30ng RNA/sample was reverse transcribed to synthesize an estimated amount of 30ng of cDNA in a reaction volume of 20uL; 20ng of cDNA/sample was amplified using the Sso Advanced SYBR Green amplification kit (Bio‐Rad). The amplified samples were diluted 1:50 for further qPCR analysis with a template volume of 1 μL per reaction.

To screen which groups of inflammatory/pain mediators were involved in the process of degeneration of the LF and IVD, a total of 80 inflammatory/pain genes (Table 2) were investigated using a custom‐made, pre‐printed 384‐well PCR array embedded with dog‐specific primers (Bio‐Rad). In addition to inflammatory mediators, markers for matrix degeneration and housekeeping genes were included (Table 2).

**TABLE 2:** Overview of the 80 inflammatory gene targets and 5 extracellular matrix genes analyzed using qPCR array grouped according to inflammatory family.

Interleukins	Interleukin receptors	Chemokines	Chemokine receptors	Other cytokines	TNF superfamily	Matrix genes
*IL1A*	*IL1R1*	*C5*	*CCR2*	*ANGPLT2*	*TNF‐α*	*ACAN*
*IL1B*	*IL1RN*	*CCL1*	*CCR3*	*BMP2*	*LTA (TNF‐β)*	*COL1a2*
*IL3*	*IL2RB*	*CCL2*	*CCR4*	*CRSP1*	*LTB (TNF‐γ)*	*COL2a1*
*IL4*	*IL2RG*	*CCL4*	*CCR5*	*CSF1*	*CD40LG*	*DCN*
*IL5*	*IL5RA*	*CCL5*	*CCR6*	*CSF2*	*FasL*	*ELN*
*IL7*	*IL6R*	*CCL7*	*CCR8*	*CSF3*	*CD70*	
*IL8*	*IL6ST*	*CCL8*	*CCR10*	*IFN‐g*	*NGF*	
*IL9*	*IL9R*	*CCL13*	*CXCR2*	*NAMPT*	*NGFR*	
*IL11*	*IL10R*	*CCL16*	*CXCR3*	*OSM*	*TNFSF10*	
*IL13*		*CCL17*	*CXCR5*	*SP*	*RANKL (TNFSF11)*	
*IL16*		*CCL20*		*SPR*	*TNFSF13*	
*IL17A*		*CCL24*		*SPP1*	*TNFSF13B*	
*IL17B*		*CCL26*		*VEGFA*		
*IL17C*		*CXCL1*				
*IL17F*		*CXCL10*				
*IL21*		*CXCL11*				
*IL27*		*CXCL12*				
*IL33*		*CXCL13*				

Abbreviations: ACAN, Aggrecan; ANGPTL2, angiopoietin‐like protein 2; BMP2, bone morphogenic protein 2; C5, complement component 5; CCL1, Chemokine (C‐C motif) ligand 1; CCR2, C‐C chemokine receptor type 2; CD40LG, cluster of differentiation 40 ligand; CD70, cluster of differentiation 70; COL1a2, Collagen type 1 alpha 2 chain; COL2a1, Collagen type 2 alpha 1 chain; CRSP1, calcitonin receptor‐stimulating peptide 1; CSF1, colony stimulating factor 1; CXCL1, Chemokine (C‐X‐C motif) ligand 1; CXCR2, C‐X‐C motif chemokine receptor 2; DCN, decorin; ELN, elastin; FasL, Fas ligand; IFN‐g, interferon gamma; IL, interleukin; IL1R1, interleukin‐1 receptor type 1; IL1RN, interleukin‐1 receptor antagonist; IL2RB, interleukin‐2 receptor subunit beta; IL2RG, interleukin‐2 receptor subunit gamma; IL5RA, interleukin‐5 receptor subunit alpha; IL6R, interleukin‐6 receptor; IL6ST, interleukin‐6 signal transducer; LTA, lymphotoxin alpha; LTB, lymphotoxin beta; NAMPT, nicotinamide phoshoribosyltransferase; NGF, nerve growth factor beta, NGFR, nerve growth factor receptor; OSM, oncostatin m; SP, substance P; SPP1, osteopontin; SPR, substance P receptor; TNF‐α, tumor necrosis factor‐alpha; TNFSF, tumor necrosis factor superfamily; VGFA, vascular endothelial growth factor a.

#### 2.5.2 Housekeeping genes

The GeNorm method embedded in the Biogazelle software package^24^, ^25^ was used to determine the optimal number and the stability of reference genes (Figure S1 and Supporting File 2). For the LF, the optimal reference gene set included *RPS5*, *RPL8*, *TATA‐binding protein (TBP)*, and *Tyrosine 3‐monooxygenase/tryptophan 5‐monooxygenase activation protein zeta (YWHAZ)*. For IVD tissue, *Succinate Dehydrogenase Complex Flavoprotein Subunit A (SDHA)*, *Ribosomal Protein S5 (RPS5)*, and *Ribosomal Protein L8 (RPL8)* were included.

#### 2.5.3 MetaCore analysis and selection of targets for additional qPCR

Gene expression of the targets embedded in the qPCR array was compared between the control and the DDD groups (*n* = 4 per group), separately for the LF and IVD tissues. The qPCR array technique allowed for quantitative analysis of the gene expression results. Relative gene expression was calculated by the efficiency corrected delta–delta Ct (ΔΔCt) method.^26^ For gene targets with a downregulation (i.e., a negative ΔΔCt value), *n*‐fold change values between 0 and 1 are generated using this methodology. To improve the display and comprehension of the magnitude of downregulation of downregulated gene targets, a negative inverse transformation (−[1/*n*‐fold change]) of *n*‐fold values between 0 and 1 was performed, generating negative *n*‐fold changes expressed in the same scale/magnitude as upregulated gene targets.

With the aim of identifying significantly regulated pathways from the large array of data, differentially expressed genes that showed an *n*‐fold regulation of <−2 or >2 were converted to their human homologs and analyzed by way of functional pathway analysis using the GeneGo MetaCore platform.^27^ The combined expression results for the LF and IVD were uploaded into the MetaCore Platform to identify significantly regulated pathways. Subsequently, gene expression of a subset of targets was further investigated by way of qPCR in a larger sample size using four additional samples of LF and IVD tissues (resulting in *n* = 8 for each target gene of interest for the control and DDD groups for both tissues). qPCR for these additional targets was performed using the Bio‐Rad CFX96 touch real‐time PCR detection system with CFX Manager Software (Bio‐Rad).

Gene expression analysis of the following tumor necrosis factor superfamily (TNFSF) signaling gene targets, identified using the qPCR array, was performed: *nerve growth factor (NGF)*, *nerve growth factor receptor (NGFR*), *tumor necrosis factor‐alpha* (*TNF‐α), lymphotoxin alpha (LTA)*, *lymphotoxin beta (LTB)*, *cluster of differentiation 40 ligand (CD40LG)*, *Fas ligand (FasL)*, *cluster of differentiation 70 (CD70)*, *tumor necrosis factor superfamily 10 (TNFSF10)*, *Receptor Activator of NF‐κB Ligand* (*RANKL/TNFSF11)*, *TNFSF13B*, and *TNFSF14*. In addition, the matrix markers *collagen 1a2* (*COL1a2)*, *COL2a1*, and *aggrecan (ACAN)* were included for gene expression analysis. As described for the array analysis, relative gene expression was calculated by the efficiency corrected delta–delta Ct (ΔΔCt) method for all evaluated genes.^26^

#### 2.5.4 WB analysis

For protein validation of the gene expression results, WB analysis for NGF and Collagen I was performed. NGF was selected as a clinically relevant component of the TNFSF superfamily and because of its known involvement in the development of discogenic pain.^28^, ^29^ Collagen I was selected as a marker for tissue degeneration/fibrosis.^30^

Four (4) control and 4 DDD LF and IVD samples were used. If possible, samples complementary to the gene expression samples were selected (Table 1; for the complete WB Protocol, see Supporting File 3). NGF and Collagen I were detected using antibodies with canine cross‐reactivity according to the manufacturer's protocol (NGF: anti‐Human NGF, LS Bio, Catalog number LS‐C388946; Collagen I: anti‐human Collagen I alpha, Novus Biologicals Catalog number NBP1‐30054). Gels were activated with Stain‐Free program using the ChemiDoc Touch (Bio‐Rad). Antibody detection was performed with ECL Western Bright (Bio‐Rad) using the ChemiDoc Touch (Bio‐Rad). Protein expression was quantified using a Chemidoc Touch System and ImageLab software (Bio‐Rad). For each lane, the total protein concentration was determined per sample using Stain‐Free Technology. The band signal for the protein of interest of each lane was subsequently normalized for the total sample protein content to determine the relative expression of each protein of interest within each sample. Stain‐Free Technology/total protein normalization was selected because of the superior protein quantitation accuracy and superior normalization reported for low abundance target proteins compared to housekeeping gene normalization.^31^, ^32^ The normalized protein expression was used as a quantitative measure for statistical analysis. Relative protein expression was calculated as the ratio of normalized protein expression (*n*‐fold change) between the DDD and control groups.

#### 2.5.5 Immunohistochemistry of RANKL

To further evaluate TNFSF signaling in the process of DDD and to validate the qPCR array results, immunohistochemistry (IHC) of RANKL (TNFSF11) was performed.

For the LF, seven control and seven DDD samples were collected from non‐chondrodystrophic dogs euthanized in non‐related experiments (DEC numbers 2007.III.08.110, 2009.III.05.037, and 2007.II.01.029) or patients affected by DLSS and requiring spinal surgery (Table 3). The LF samples were classified as normal or degenerated/hypertrophic as defined above and collected using similar methods.

**TABLE 3:** Overview of patient samples (number, breed, age, gender, collected tissue, and status) used for immunohistochemistry of RANKL.

Ligamentum flavum samples				
Sample nr.	Breed	Age	Gender	Status
1	Beagle	1y 10m	F	Control
2	Beagle	2yj 2m	F	Control
3	Beagle	2y 11m	F	Control
4	Beagle	3y 2m	F	Control
5	German Shepherd Dog	9y 9m	FC	Control
6	Labrador Retriever	8y 6m	M	Control
7	Mixed breed	11y 10m	MC	Control
8	German Shepherd Dog	5y 10m	M	DDD
9	Newfoundlander	3y 3m	FC	DDD
10	White Shepherd Dog	5y 10m	M	DDD
11	German Shepherd Dog	5y 10m	M	DDD
12	Rhodesian Ridgeback	11y 0m	MC	DDD
13	Malinois	10y 0m	M	DDD
14	Mixed Breed	6y 2m	FC	DDD

Intervertebral disc samples, NCD				
Sample nr.	Breed	Age	Gender	Thompson grade
1	Mixed breed	1y 4m	F	1
2	Flatcoated retriever	7y	F	1
3	Mixed breed	1y 5m	F	1
4	Kerry beagle	3y	M	1
5	Bernernese Mountain Dog	10y 1m	MC	1
6	Great Dane	5y 5m	MC	1
7	Mixed breed	1y 4m	F	2
8	Foxhound	7y 2	F	2
9	Foxhound	7y 2m	F	2
10	Mixed breed	4y 2m	F	2
11	Mongrel	3y 4m	F	2
12	Labrador	11y 0m	M	2
13	Foxhound	7y 0m	F	3
14	Foxhound	10y	M	3
15	Foxhound	10y	M	3
16	Bouvier des Flandres	13y 10m	M	3
17	Mixed breed	5y 1m	F	3
18	Mixed breed	10y	M	3
19	Foxhound	7y	F	4
20	Foxhound	9y	F	4
21	Bernese Mountain Dog	10y	MC	4
22	Mixed breed	10y 6m	MC	4
23	Welsh Terrier	16y	M	5
24	Irish Terrier	13y	M	5
25	Labrador Retriever	11y 2m	M	5
26	Labrador Retriever	8y 4m	M	5
27	German Shepherd Dog	9y 6m	FC	5

Intervertebral disc samples, CD				
Sample nr.	Breed	Age	Gender	Thompson grade
1	Beagle	2y 1m	M	1
2	Beagle	3y	F	1
3	Beagle	3y	F	1
4	Beagle	2y 10m	F	1
5	Beagle	2y 10m	F	1
6	Beagle	3y 1m	F	1
7	Beagle	2y 1m	F	2
8	Beagle	2y 4m	F	2
9	Beagle	2y 1m	F	2
10	Beagle	2y 1m	F	2
11	Beagle	2y 1m	M	2
12	Beagle	2y 1m	M	2
13	Beagle	9y 9m	F	3
14	Beagle	2y 1m	F	3
15	Beagle	2y 1m	M	3
16	Beagle	2y 2m	M	3
17	Beagle	3y 1m	F	3
18	Beagle	8y 2m	F	4
19	Beagle	9y 7m	F	4
20	Beagle	10y	F	4
21	Beagle	10y	F	4
22	Beagle	8y 2m	F	5
23	Beagle	9y 7m	F	5
24	Beagle	10y	F	5

Abbreviations: CD, chondrodystrophic; DDD, degenerative disc disease; F, female; FC, female castrated; M, male; MC, male castrated; NCD, non‐chondrodystrophic.

Complete IVDs (endplate‐disc‐endplate) were collected from both chondrodystrophic and non‐chondrodystrophic dogs euthanized in non‐related experiments (same DEC numbers above). These animals did not show clinical signs of degenerative spinal disease. IVD samples were divided into five different grades of degeneration based on gross morphology of midsagittal sections, ranging from Thompson grade I for healthy to Thompson grade V for end‐stage degeneration (*n* = 12, 12, 11, 8, and 8 for grades I–V, respectively; Table 3).^33^

Five‐micrometer‐thick sections of each tissue sample were mounted on KP+ microscope slides and processed for RANKL IHC (primary antibody: Abcam ab216484; IHC protocol: Supporting File 4). Positive control tissues included dog lymph node tissue.^34^ Negative controls were included by omitting the primary antibody. IHC‐stained slides were scanned using a Nanozoomer 2.0HT slide scanner (Hamamatsu Photonics). Adobe Photoshop. Hamamatsu NDPI view software was used to manually count positively stained RANKL cell numbers in three randomly selected LF areas per slide. Similarly, for the IVD samples, positively stained RANKL cell numbers were counted in four randomly selected regions separately for the nucleus pulposus, dorsal AF, and ventral AF. The mean percentage of positive cells/total cells as well as the localization (nuclear, cytoplasmic, and membranous) of positive staining were determined for each section in each sample.

### 2.6 Statistical analysis

Statistical analyses were performed using R statistical software.^35^ Linear mixed models, containing both fixed and random effects,^36^ were used to analyze the described parameters for the qPCR of tissue samples, WB analysis, and IHC. The Akaike information criterion (AIC) was used for model selection. A random intercept for each dog was added to each model to take the correlation of the observations within a dog into account. If necessary, models were optimized by correcting for unequal variances and/or for autoregressive correlation. Conditions for the use of mixed models, including normal distribution of the data, were assessed by analyzing the residuals (PP and QQ plots) of the acquired models; no violations of these conditions were observed.

For the qPCR analysis of LF and IVD samples, the ΔCT for individual target genes was used as a parameter value. For the WB analysis, the signal intensity normalized for the total protein content of each line was used as a parameter value. For both qPCR and WB analysis, the explanatory factors for the linear mixed model were “Tissue” (LF and IVD), “degeneration stage” (control vs. DDD group), and the interaction between these factors. For IHC analysis, log‐transformation of the percentages was performed to attain normal distribution of the data. The percentage of positive cells per section was used as a parameter value. The explanatory factors for the linear mixed model were “Tissue” (LF, NP, dorsal AF, and ventral AF), “degeneration stage” (control vs. DDD group for the LF, and Thompson grades I–V for the IVD), and the interaction between these factors.

*p* Values were calculated per target gene to analyze differences between tissues and degeneration stages. For all the above‐described models, the Benjamini‐Hochberg correction was used to correct for multiple comparisons. *p* < 0.05 was considered statistically significant.

## 3 RESULTS

### 3.1 qPCR array and MetaCore analysis

#### 3.1.1 Matrix markers

**FIGURE 1:**
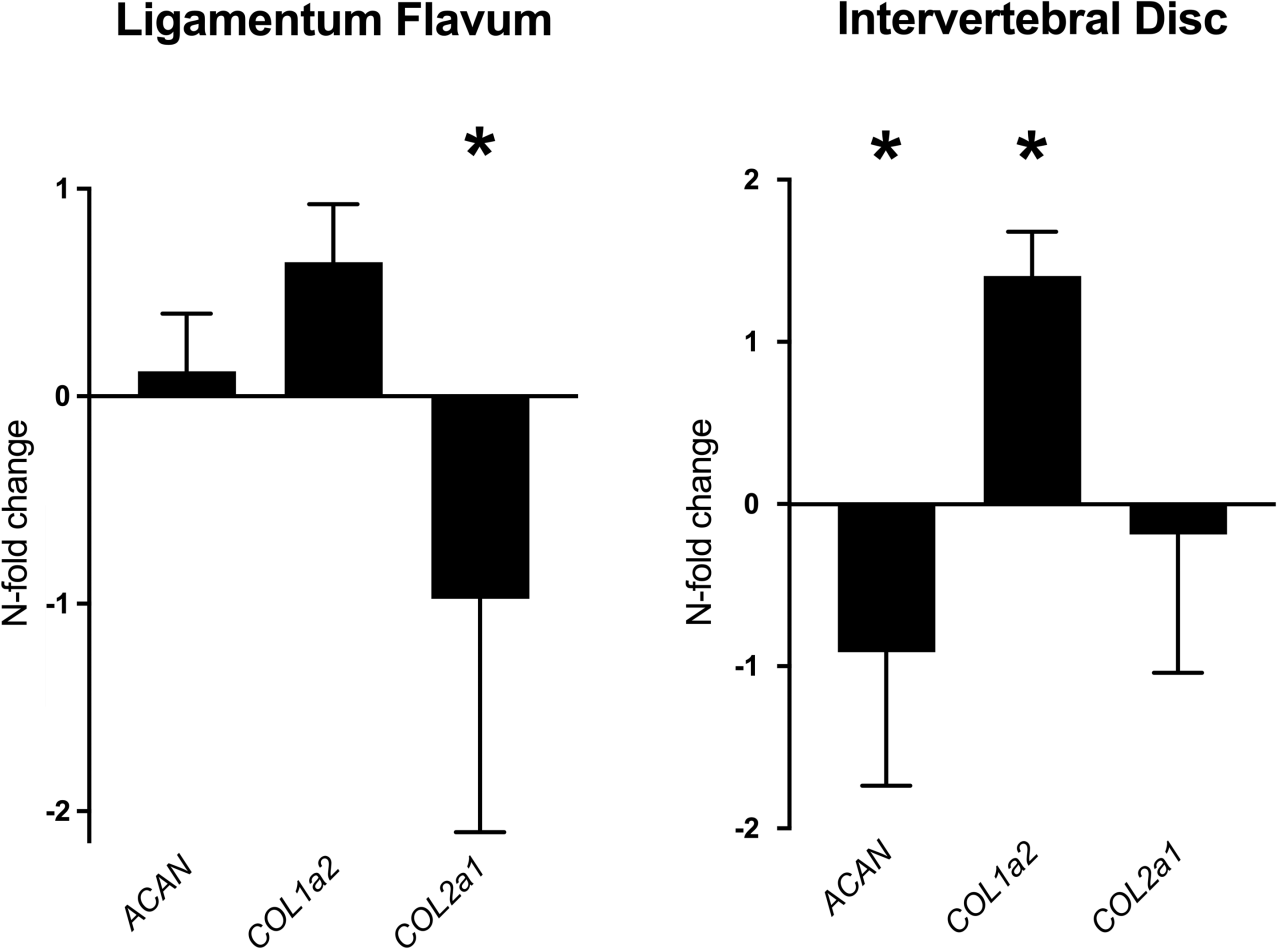
*N*‐fold changes (mean ± SD) in gene expression of extracellular matrix targets (*X*‐axis) for the ligamentum flavum and intervertebral disc. The fold changes indicate the relative expression of the DDD group relative to the control group. For display purposes, *n*‐fold changes (Table 4) were transformed using a log transformation. The *Y*‐axis values represent the *n*‐fold changes after log transformation. ACAN, aggrecan; COL1a2, Collagen I; COL2a1, Collagen II; DDD, degenerative disc disease. *=*p* < 0.01.

LF and IVD tissues showed differential expression for the evaluated matrix gene targets (Figure 1). For the LF, only the expression of *COL2a1* was significantly downregulated in the DDD group compared to the control group (mean *n*‐fold change ± SD: −9.5 ± 10.3; *p* = 0.020). For the IVD, the gene expression of *ACAN* was significantly downregulated (−8.2 ± 9.7; *p* = 0.004) and *Col1a2* gene expression was significantly upregulated (25.2 ± 22.2; *p* = 0.001) in the DDD group. These changes in matrix marker expression verify the degenerative state of the DDD group for both the LF and IVD tissues.

#### 3.1.2 Inflammatory gene targets

A total of 30 and 41 inflammation/pain‐related genes embedded in the qPCR array were significantly regulated for the LF and IVD, respectively (Table 4). For the LF, all significantly regulated genes showed a downregulation. Conversely, for the IVD, 30 of the 41 significantly regulated genes were upregulated.

**TABLE 4:** Significantly regulated inflammatory genes identified in the qPCR array analysis, showing means ± SD for the *n*‐fold changes for the ligamentum flavum (LF) and intervertebral disc (IVD) for the different cytokine groups.

Ligamentum flavum				Intervertebral disc			
	*N*‐fold	SD	*p*		*N*‐fold	SD	*p*
**Tumor necrosis factor superfamily members**							
***NGF***	**−18.8**	**21.3**	**0.001**	***NGF***	**−29.8**	**24.9**	**0.000**
*NGFR*	−12.4	16.1	0.040	*CD40LG*	142.1	138.7	0.049
*LTA*	−33.1	46.4	0.018	*CD70*	80.6	70.1	0.007
*LTB*	−19.5	23.2	0.001	*TNFSF10*	2.7	4.8	0.014
*FASLG*	−293.9	346.6	0.000	*TNFSF11*	10.1	8.7	0.000
**Interleukins and receptors**							
***IL2***	**−580.4**	**678.1**	**0.000**	***IL2***	**−81.2**	**54.3**	**0.002**
*IL3*	−3.8	0.0	0.017	*IL4*	−57.0	35.4	0.000
*IL11*	−18.1	21.7	0.001	*IL6*	172.6	118.4	0.000
*IL13*	−20.7	27.4	0.024	*IL7*	5.8	4.1	0.020
*IL17C*	*	*	*	*IL15*	2.1	3.9	0.016
*IL21*	*	*	*	*IL17A*	*	*	*
*IL2RB*	−54.8	61.7	0.000	*IL21*	−28.1	17.2	0.000
*IL6R*	−26.1	29.0	0.002	*IL33*	62.0	49.1	0.001
				*IL1R1*	2.9	4.5	0.000
				*IL1RN*	15.7	11.7	0.002
				*IL2RG*	5.6	3.7	0.000
				*IL6ST*	2.1	4.4	0.016
				*IL10RA*	21.4	15.4	0.000
**Chemokines and receptors**							
***CXCL1***	**−5.6**	**6.8**	**0.049**	*C5*	1578.1	1493.7	0.002
*CXCL10*	−8.7	10.7	0.049	*CCL1*	57.4	53.4	0.025
***CCR2***	**−14.4**	**16.2**	**0.001**	*CCL2*	18.0	14.1	0.002
*CCR3*	−101.3	137.8	0.008	*CCL4*	6.8	5.5	0.002
*CCR4*	−37.5	45.0	0.000	*CCL5*	8.3	7.0	0.013
***CCR5***	**−12.2**	**13.3**	**0.011**	*CCL8*	4.3	3.0	0.001
*CCR8*	−53.5	0.0	0.000	*CCL17*	601.6	500.4	0.014
*CXCR2*	−43.7	53.2	0.018	*CCL24*	6.1	4.3	0.006
*CXCR3*	−38.2	52.7	0.005	***CXCL1***	**5.6**	**3.7**	**0.001**
***CXCR5***	**−20.8**	**24.8**	**0.010**	*CXCL12*	18.1	13.2	0.000
				*CXCL13*	138.7	123.8	0.011
				***CCR2***	**57.3**	**41.0**	**0.048**
				***CCR5***	**7.4**	**5.6**	**0.008**
				*CCR10*	−4.3	3.3	0.000
				***CXCR5***	**−7.1**	**4.7**	**0.000**
**Other cytokines and receptors**							
***CRSP1***	**−55.9**	**63.1**	**0.003**	*ANGPTL2*	5.6	4.1	0.000
***CSF2***	**−19.6**	**22.1**	**0.030**	*BMP2*	−6.4	5.1	0.000
*CSF3*	−29.4	34.7	0.000	***CRSP1***	**−16.3**	**11.0**	**0.010**
*OSM*	−39.3	44.9	0.000	*CSF1*	2.8	4.2	0.000
*SP*	−367.5	443.4	0.000	***CSF2***	**−3.7**	**2.5**	**0.000**
*SPR*	−8.7	11.3	0.028	*IF‐Gamma*	733.3	636.4	0.038
***SPP1***	**−13.2**	**16.6**	**0.006**	***SPP1***	**−3.1**	**4.6**	**0.008**
				*VEGFA*	−14.4	12.2	0.009
**Matrix genes**							
*COL2A1*	−9.5	10.3	0.020	*ACAN*	−8.2	9.6	0.004
				*COL1A2*	25.5	22.2	0.001

*Note*: Targets that were significantly regulated for both the LF and IVD have been highlighted in bold text. For abbreviations, see the legend of Table 2.

* indicates that gene expression was only found in samples of the control group.

When performing a combined MetaCore pathway analysis of LF and IVD tissues, a total of 230 significantly regulated inflammatory and pain‐related signaling pathways were identified. Pathways of particular interest that were significantly regulated in both tissues included *TNF‐induced inflammatory signaling and nuclear factor kappa‐light‐chain‐enhancer of activated B cells (NF‐kB)* and *Mitogen‐activated protein kinases (MAPKs)‐mediated pro‐inflammatory cytokine production*. Moreover, different members of the *TNFSF* were identified and were differentially expressed between LF and IVD tissues (Table 4). These results combined prompted us to select the TNFSF for further analysis.

### 3.2 qPCR analysis of TNFSF members

**FIGURE 2:**
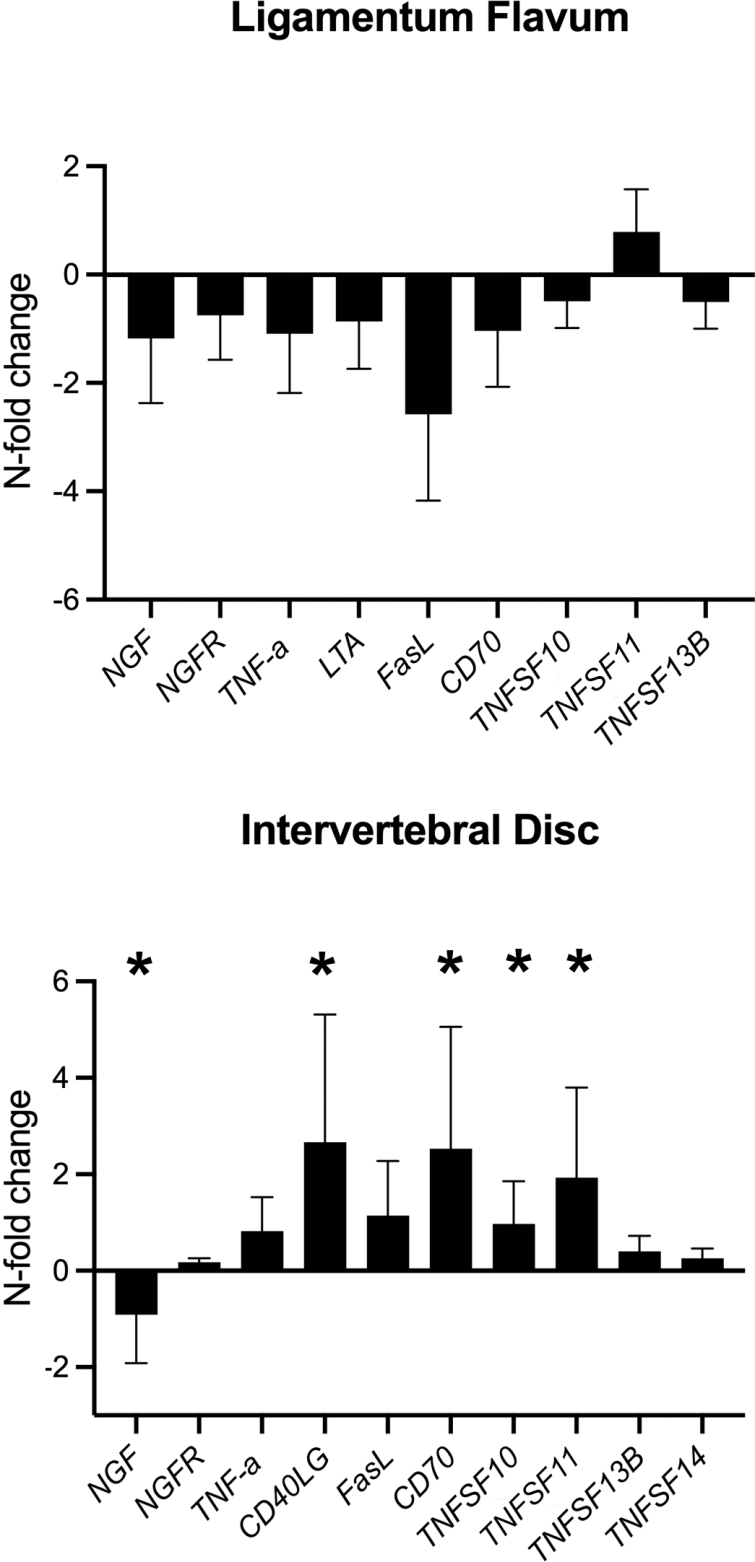
*N*‐fold changes (mean ± SD) in gene expression of the evaluated tumor necrosis factor superfamily members (*X*‐axis) involved in degeneration of the ligamentum flavum and intervertebral disc. The fold changes indicate the relative expression of the DDD group relative to the control group. For display purposes, *n*‐fold changes (Table 5) were transformed using a log transformation. The *Y*‐axis values represent the *n*‐fold changes after log transformation. *=*p* < 0.01. DDD, degenerative disc disease.

For the LF, no significant differences between the control and DDD groups for any of the analyzed targets were found (Figure 2, Table 5). Overall, a high degree of variance in gene expression was observed for all targets.

**TABLE 5:** Means ± SD for the dCT values and *n*‐fold change (DDD vs. control group) and associated *p* values obtained for the different gene targets involved in TNFSF signaling for the ligamentum flavum and the intervertebral disc.

Target	Healthy	Diseased	*N*‐fold change	*p* Value
**Ligamentum flavum**				
*NGF*	5.74 ± 2.43	9.64 ± 5.28	−15.0 ± 15.4	0.146
*NGFR*	6.21 ± 2.50	8.69 ± 2.76	−5.6 ± 6.6	0.369
*TNF‐α*	8.41 ± 8.12	12.04 ± 7.68	−12.3 ± 12.4	0.532
*LTA*	12.08 ± 7.52	14.94 ± 5.21	−7.3 ± 7.5	0.599
*LTB*	NE	NE	NE	
*CD40LG*	NE	NE	NE	
*FasL*	7.63 ± 7.41	12.91 ± 6.14	−38.6 ± 39.1	0.580
*CD70*	9.49 ± 8.97	12.93 ± 7.63	−10.8 ± 10.9	0.302
*TNFSF10*	2.89 ± 4.18	4.53 ± 8.90	−3.1 ± 3.1	0.147
*TNFSF11*	11.03 ± 6.11	8.41 ± 8.35	6.2 ± 6.1	0.752
*TNFSF13B*	5.82 ± 6.99	7.44 ± 7.49	−3.2 ± 3.1	0.539
*TNFSF14*	NE	NE	NE	
**Intervertebral disc**				
*NGF*	3.98 ± 3.05	7.01 ± 2.31	−8.1 ± 10.2	**0.001**
*NGFR*	9.98 ± 2.95	9.41 ± 2.32	1.5 ± 1.2	0.729
*TNF‐α*	12.96 ± 4.56	10.24 ± 2.19	6.6 ± 5.1	0.275
*LTA*	NE	NE	NE	
*LTB*	NE	NE	NE	
*CD40LG*	21.31 ± 5.77	12.45 ± 4.41	464.2 ± 442.3	**0.002**
*FasL*	17.54 ± 5.34	13.73 ± 5.05	14.0 ± 13.6	0.288
*CD70*	18.23 ± 4.32	9.81 ± 6.02	341.5 ± 336.3	**0.003**
*TNFSF10*	5.49 ± 3.20	2.25 ± 2.45	9.4 ± 7.7	**0.003**
*TNFSF11*	11.48 ± 4.50	5.07 ± 2.86	85.3 ± 73.6	**0.002**
*TNFSF13B*	5.13 ± 3.19	3.83 ± 2.80	2.5 ± 2.1	0.292
*TNFSF14*	16.75 ± 5.92	15.93 ± 3.56	1.8 ± 1.6	0.718

*Note*: All *n*‐fold changes have been expressed as fold increased or decreased expression relative to the healthy group. Significant changes (*p* < 0.05) have been highlighted in bold text. NE indicates that no evident expression was found in the samples analyzed.

Abbreviations: DDD, degenerative disc disease; TNFSF, tumor necrosis factor superfamily.

For the IVD, gene expression of *CD40LG* (mean *n*‐fold change ± SD: 464 ± 442; *p* = 0.002), *CD70* (341 ± 336; *p* = 0.003), *TNFSF10* (9.4 ± 7.7; *p* = 0.003), and *TNFSF11* (85 ± 74; *p* = 0.002) was significantly higher in the DDD compared to the control group (Figure 2, Table 5). The gene expression of *NGF* was significantly lower (−8.1 ± 10; *p* = 0.001) in the DDD compared to the control group, while the expression of *NGFR*, *TNF‐α*, *FasL*, *TNFSF13B*, and *TNFSF14* was comparable to control tissues. *LTA* and *LTB* were undetectable.

### 3.3 WB analysis

**FIGURE 3:**
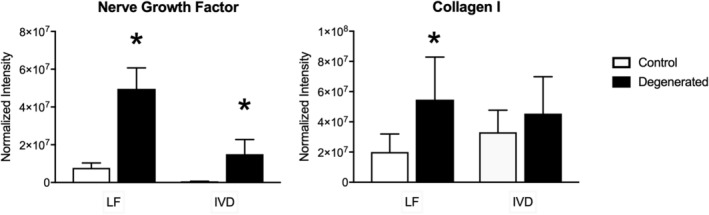
Western blot results for the proteins nerve growth factor and Collagen I, showing the protein expression intensity normalized for the total protein content (normalized intensity) for the healthy and degenerated ligamentum flavum (LF) and intervertebral disc (IVD). *=*p* < 0.05.

**FIGURE 4:**
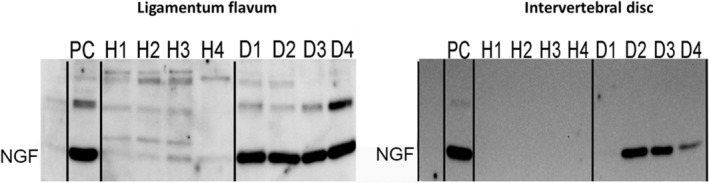
Typical example of western blot results for nerve growth factor (NGF) expression of healthy (H) and degenerated (D) ligamentum flavum and intervertebral disc. PC, positive control. The expression in the degenerated group was significantly higher compared to the healthy group.

Protein expression of NGF was significantly higher (mean *n*‐fold change ± SD) in the DDD compared to the control group for both the LF (4.4 ± 0.5; *p* < 0.0001) and the IVD (11.3 ± 5.6; *p* = 0.003; Figures 3 and 4). Furthermore, protein expression for Collagen 1 was significantly higher in the degenerated LF compared to the control group (1.3 ± 0.4; *p* = 0.045). For the IVD, protein expression of Collagen I was comparable between the DDD and control groups (*p* = 0.394).

### 3.4 Immunohistochemistry for RANKL

**FIGURE 5:**
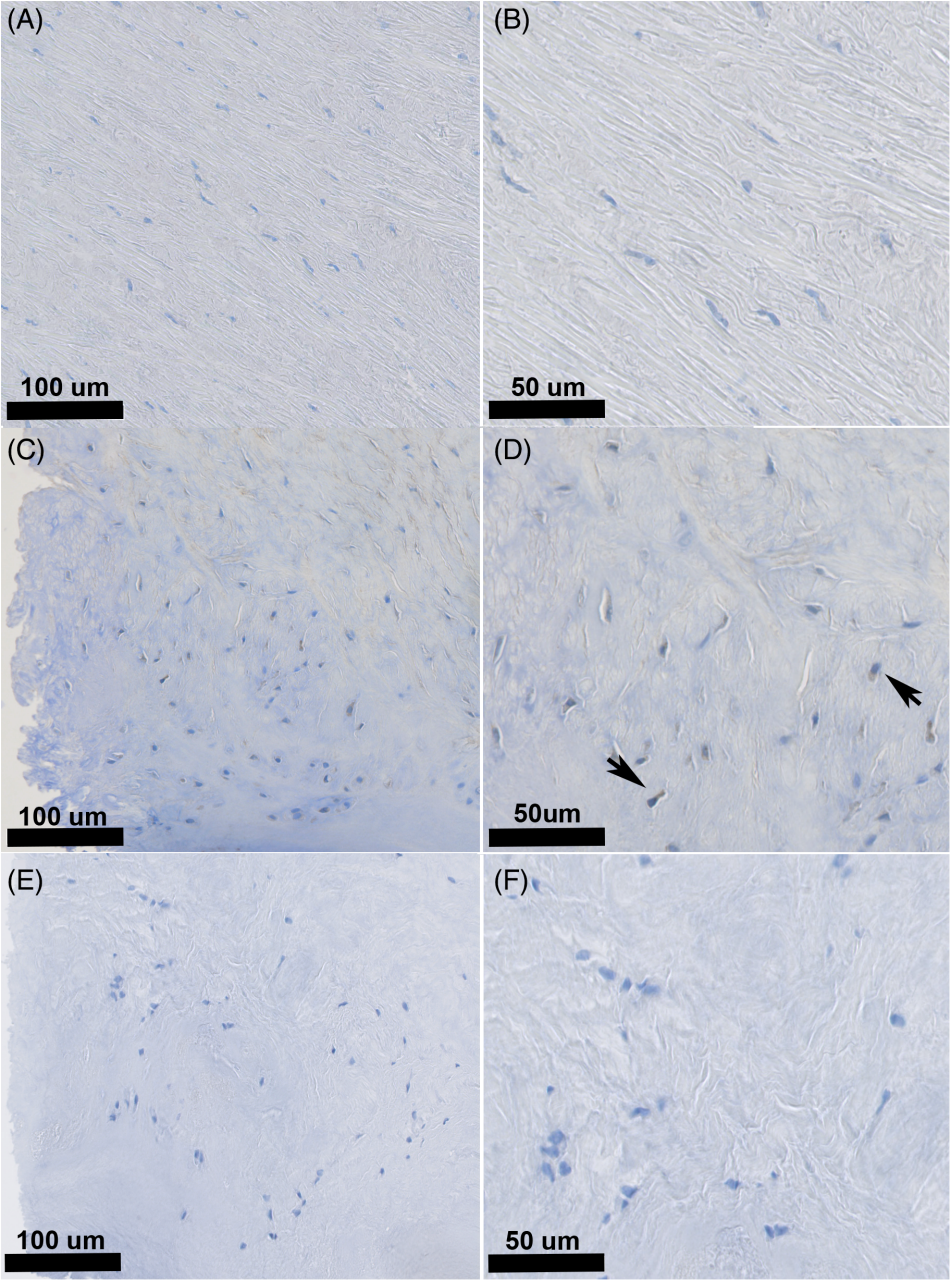
Histological slides of ligamentum flavum (LF) for the control (A, B) and DDD (C, D) groups immunohistochemically stained for RANKL. LF cells were largely negative for RANKL protein expression, with occasional positively stained cells (arrowheads). RANKL protein expression was mainly found to be cytoplasmatic. Negative control slides of ligamentum flavum of the DDD group (E, F) corresponding to slides C and D showed no RANKL immunopositivity. DDD, degenerative disc disease.

For the LF, RANKL immunopositivity was comparable between the control and DDD groups (*p* = 0.13; Table 6). For both groups, the majority of the LF cells were negative, with a small percentage (control group: 0%–14.0%; DDD group: 0%–23.0%) of cells showing cytoplasmic immunopositivity (Figure 5).

**TABLE 6:** Median and ranges for percentage of cells positively stained for RANKL in ligamentum flavum (LF), nucleus pulposus (NP), dorsal annulus fibrosus (AF), and ventral AF tissue.

Tissue	Group	Median (%)	Range (%)
LF	control	1.79	0–14
	DDD	7.08	0–23
NP	Thompson I	0	0–4.0
	Thompson II	0	0–28.0
	Thompson III	1.7	0–36.0
	Thompson IV	27.7*	6.0–59.0*
	Thompson V	75.0*	37.0–76.0*
Dorsal AF	Thompson I	0	0–0.0
	Thompson II	0	0–4.0
	Thompson III	0	0–8.0
	Thompson IV	0	0–26.0
	Thompson V	27.1*	0–55.0*
Ventral AF	Thompson I	0	0–0
	Thompson II	0	0–5.0
	Thompson III	0	0–9.0
	Thompson IV	0	0–0
	Thompson V	25*	17.0–19.0*

*Note*: For the LF, control and degenerated (DDD) tissues were evaluated, whereas for the intervertebral disc tissues, Thompson grade I (non‐degenerated) to grade V (end‐stage degeneration) were evaluated.

Abbreviation: DDD, degenerative disc disease.

* indicates a significant difference with all other degeneration groups (*p* < 0.05).

**FIGURE 6:**
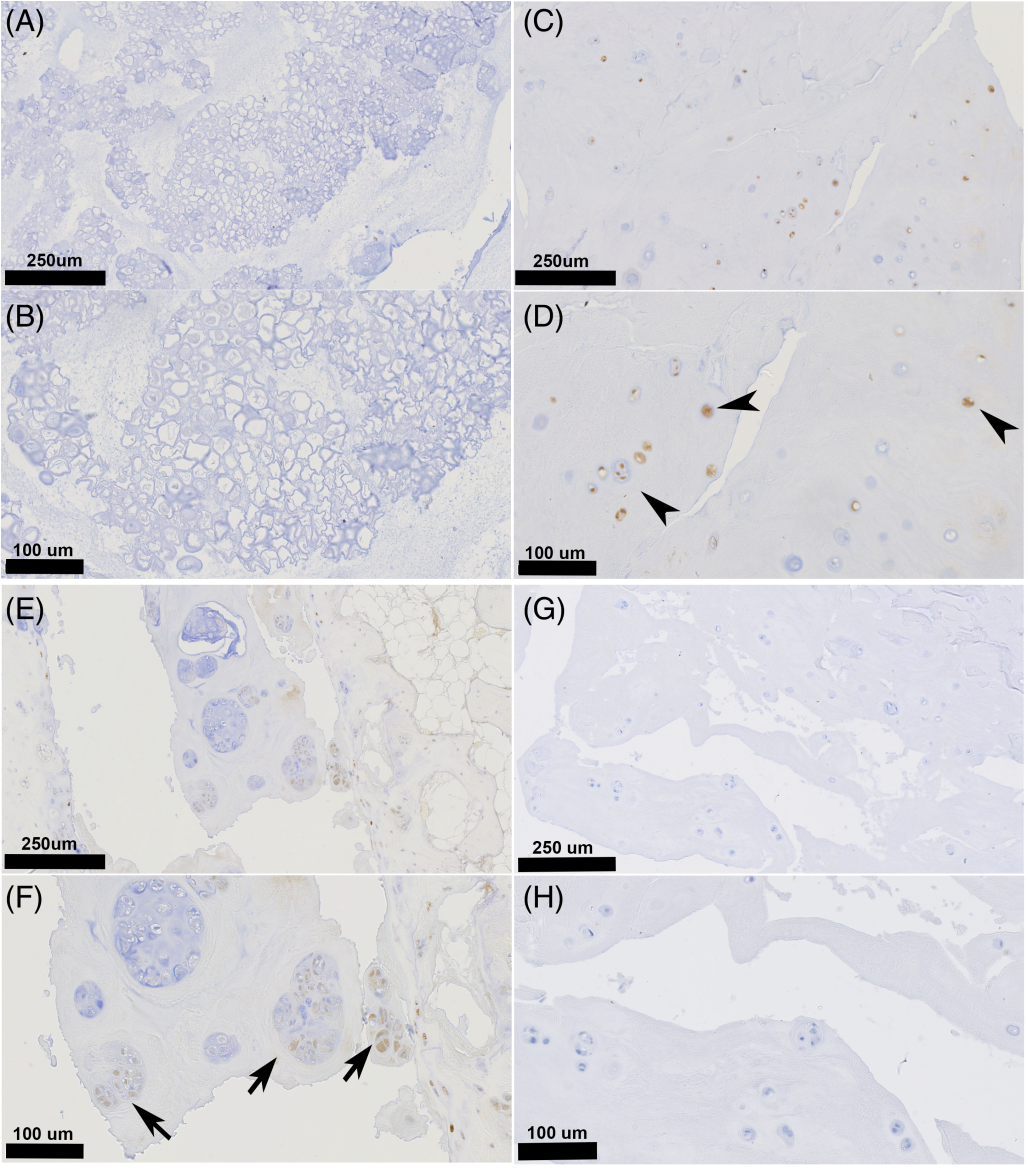
Histological slides of nucleus pulposus for Thompson grade I (A, B), Thompson grade IV (C, D), and Thompson grade V (E, F) immunohistochemically stained for RANKL. For Thompson grade I, all NP cells were negative for RANKL (A, B). With progressive chondrification of the NP, individual chondrocyte‐like cells showed increasingly more membranous and cytoplasmatic RANKL protein expression (C, D, arrowheads). For end‐stage NP tissue, chondrocyte‐cell nests expressing a high proportion of RANKL protein could be observed (E, F, arrows). Negative control slides (G, H) of nucleus pulposus for Thompson grade IV (corresponding to slides C and D) showed no immunopositivity. NP, nucleus pulposus.

In contrast, IVD degeneration involved a significant increase in RANKL immunopositivity. In the NP, notochordal cells and chondrocyte‐like cells were largely negative for RANKL in Thompson grades I (median % positive cell: 0%), II (0%), and III (1.7%) discs (Figure 6, Table 6, Table S1). Progressively higher RANKL immunopositivity was found for Thompson grades IV (27.7%; *p* ≤ 0.019) and V discs (75.0%; *p* < 0.001), with the cells mainly showing cytoplasmic staining.

**FIGURE 7:**
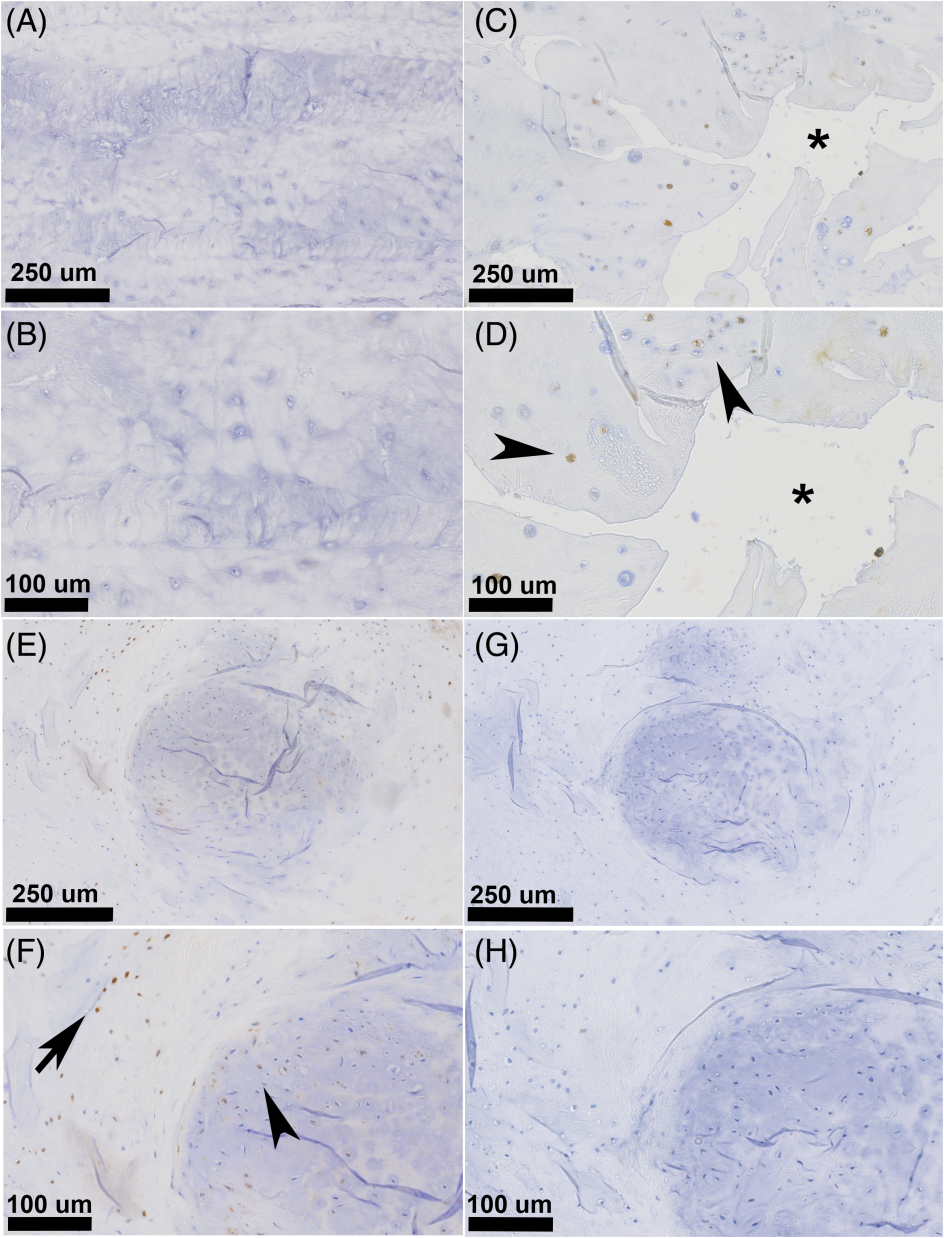
Histological slides of annulus fibrosus (AF) for Thompson grade I (A, B) and Thompson grade V (C–F) immunohistochemically stained for RANKL. For Thompson Grade I, the AF has a normal lamellar architecture with all AF cells negative for RANKL. With progressive chondrification and structural disorganization of the AF, individual AF cells showed increasingly more cytoplasmatic RANKL protein expression (arrowheads), especially cells surrounding structural AF defects (*). Occasionally, large collections of chondroid cells were found in outer AF layers, showing clear RANKL protein expression (arrows). Negative control slides (G, H) of AF for Thompson grade V (corresponding to slides E and F) showed no immunopositivity.

Similarly, progressive degeneration and chondrification of the AF involved a significant increase in RANKL immunopositivity. In the dorsal and ventral AF, Thompson grades I–IV discs were largely negative for RANKL, while Thompson grade V discs showed significantly higher RANKL expression compared to earlier degeneration stages (*p* < 0.034; Figure 7). Subjectively, positive RANKL immunostaining was mainly evident in cells surrounding annular clefts or tears. In occasional samples (*n* = 2), large accumulations of chondrocyte‐like cells within the dorsal AF could be observed, with these cells and surrounding AF cells showing clear RANKL immunopositivity.

The expression profile of RANKL did not differ between chondrodystrophic and non‐chondrodystrophic IVDs.

## 4 DISCUSSION

This is the first study to screen a large array of inflammatory and pain‐related mediators in the LF and IVD of dogs suffering from naturally occurring DDD. Degeneration of the LF and IVD involved the regulation of various inflammatory and pain‐related signaling mediators, including members of the TNFSF. While *CD40LG*, *CD70*, *TNFSF10*, NGF, and RANKL/TNFSF11 were upregulated in the degenerated IVD, significant upregulation in NGF expression in the LF was not accompanied by alterations in the gene expression of the studied TNFSF members. These findings suggest that multiple TNFSF ligands are involved in the degenerative cascade of the IVD, while in the LF, apart from NGF, other biomolecular signaling pathways may be at play.

### 4.1 Interleukins, chemokines, and other inflammatory mediators

Several interleukins, chemokines, and other inflammatory mediators differentially expressed in the process of DDD were identified using qPCR array. Apart from the TNFSF, the main inflammatory groups studied were interleukins and associated receptors, chemokines and associated receptors, and other inflammatory mediators.

Specifically for the LF, the expression of IL‐1, IL‐6, IL‐8, and IL‐15 has been positively correlated to the thickness of hypertrophied LF tissue in people^30^ and IL‐6 has been shown to promote osteogenic differentiation of LF cells.^37^ Similarly, increased expression of the chemokines *CX3CL1* and *CX3CR1* has been associated with LF hypertrophy in humans.^38^ In contrast to these findings, the gene expression of ILs, chemokines, or other cytokines was not increased in the degenerated LF in the present study. This discrepancy may reflect differences in degeneration stage between studies and pathomechanistic differences in LF degeneration between humans and dogs.

In contrast, for the IVD several interleukins were significantly regulated in the present work, including an upregulation of *IL‐6*, *IL‐7*, and *IL‐15* and a downregulation of *IL‐2*, *IL‐4*, and *IL‐21*. Differential expression of these *ILs* has also been identified in human IVD degeneration,^15^, ^39^ and as such it appears that IVD degeneration in dogs involves similar inflammatory/catabolic mediators, further establishing the dog as a model for human DDD. Apart from ILs, the current study identified several chemokines and other cytokines significantly regulated in the process of IVD degeneration in dogs, including *CCL2*, *CCL4*, *CCL5*, *CCL8*, *CCL24*, *CXCL12*, *CXCL13*, *CCR2*, *ANGPTL2*, and *SPP1*. These specific chemokines have also been reported in other studies investigating naturally occurring canine^7^, ^40^ and (painful) human^41^, ^42^, ^43^, ^44^, ^45^, ^46^, ^47^, ^48^, ^49^, ^50^, ^51^ IVD degeneration. Interestingly, several of these factors have been associated with MCs^52^ and nerve sprouting within the IVD tissue resulting in discogenic pain.^42^, ^45^, ^47^

Apart from previously reported inflammatory mediators, the present qPCR array also revealed previously unidentified ILs, chemokines, and other mediators regulated in degenerating IVD tissue, including an upregulation of *IL‐33*, *CCL17*, and *CSF1*, and a downregulation of *CCR10*, *CXCR5*, *CRSP1*, and *CSF2*. These results indicate that the degenerative cascade within the IVD involves a complex array of inflammatory mediators that still remain largely unexplored. However, it should be noted that all above‐discussed mediators identified using the qPCR array were investigated on a gene expression level using small sample population. Hence, although these significantly regulated gene targets appear interesting, they require further investigation in a large sample size and should be validated on a protein level.

### 4.2 TNFSF members and degenerative spinal disease

The TNFSF is a family of inflammatory mediators that regulates cell proliferation, cell death, and morphogenesis.^53^ TNF superfamily receptors are known to mediate various types of cancer and immune‐mediated diseases.^53^ As such, stimulating or inhibiting TNFSF signaling pathways is expected to have therapeutic benefits for patients affected by such diseases.^53^ In contrast, the regulation of the TNFSF in joint/spinal disease has been less well explored.^54^, ^55^, ^56^ Of all the different TNFSF members, the most commonly investigated is TNF‐α. Increased expression of TNF‐α has been suggested to play a leading role in the ossification of LF^57^ and in IVD degeneration in people.^14^, ^58^, ^59^, ^60^, ^61^, ^62^ Similarly, a more recent study reported increased levels of TNF‐α on a gene and protein level in dogs suffering from DDD.^63^ In the current study, no significant regulation in *TNF‐α* expression was found for both the LF and IVD. One explanation for this discrepancy may be fundamental differences in the degenerative/inflammatory cascade between the reported studies and the present one: in the study by Monchaux et al.,^63^ IVD material was collected from dogs suffering from IVD extrusion in the thoracolumbar spine. In contrast, in the present study, LF and IVD tissues were collected from dogs suffering from lumbosacral DDD, involving LF hypertrophy without ossification and IVD prolapse.

Apposed to TNF‐α, several other TNFSF members were significantly regulated in the process of DDD, primarily at the IVD level and not in the neighboring LF. Although the regulated TNFSF members have been reported in the human literature as individual agents in DDD, the current work is the first to report the differential regulation of multiple TNFSF ligands in tissue from the same dog suffering from naturally occurring DDD. The regulation of these different TNFSF members may have several different cellular/biomolecular implications.

The TNFSF ligand CD40LG acts as a ligand for CD40 receptor for activation of CD40‐CD40LG signaling with effects depending on cell types, such as B‐cell activation, NF‐kB signaling, and anti‐apoptotic signaling.^64^ The regulation of NF‐kB signaling through CD40LG has been implicated to be involved in IVD degeneration in humans.^65^ To our knowledge, the present study is the first to show that *CD40* expression is significantly upregulated in the degenerated IVD in a model of spontaneous IVD degeneration and as such, may implicate an activation of NF‐kB signaling in naturally occurring DDD.

In addition to *CD40LG*, *CD70* was significantly upregulated in the degenerated IVD in dogs. CD70 is a type II transmembrane glycoprotein, known to be expressed in B cells, T cells, mast cells, NK cells, and activated dendritic cells.^66^ Specifically, for the IVD, a population of NP progenitor cells positive for CD70 has recently been identified through RNA‐Seq analysis.^67^ Hence, it may be postulated that the upregulation of *CD70* found here represents a recruitment or proliferation of this particular progenitor population in the process of DDD.

TNFSF10 or TNF‐related apoptosis‐inducing ligand (TRAIL) is known to induce apoptosis in various cell types in a caspase‐dependent manner.^66^ In the IVD, the caspase system can be activated through extrinsic death receptors (DRs), mitochondrial (intrinsic), and endoplasmic reticulum pathways.^68^ These three pathways play different roles in different stages of the degenerative cascade, where the extrinsic and endoplasmic reticulum pathways play a major role in mild IVD degeneration, whereas the mitochondrial pathway appears to play a major role in moderate and severe IVD degeneration.^69^, ^70^ In the exogenous DR pathway, death ligands interact with cell surface DRs to induce the generation of DR signaling platforms, thereby triggering the initiation of apoptosis.^71^ The major DRs include tumor necrosis factor‐receptor‐1 (TNFR1), Fas, and tumor necrosis factor‐related apoptosis‐inducing ligand receptor (TRAILR).^72^ TRAIL binds to these receptors to activate the extrinsic apoptosis signaling pathway by forming a death‐inducing signaling complex (DISC). Specifically for the IVD, DR4 and DR5 are two types of TRAILR that are important molecular mediators in the induction of apoptosis and the expression of the TRAIL/DR4/DR5 axis components has been reported in human IVD cells.^52^ Gene polymorphism of TRAIL is associated with IVD degeneration, and the expression of TRAIL is positively correlated with the degeneration grade of the IVD.^52^ Moreover, the gene expression of *caspase 8*, which is a downstream activator caspase of TRAIL, has been significantly correlated with the progression of IVD degeneration.^52^

The activation of apoptosis through TRAIL appears to be subject to regulation through miRNAs: the upregulation of miRNA‐181, which directly targets TRAIL, has been shown to inhibit TRAIL and thereby to exert anti‐apoptotic effects.^73^ Also, the inhibition of TRAIL by miRNA‐98 could result in a downregulation of caspases 8 and 3 expression to inhibit apoptosis of IVD cells.^74^

Therefore, it is tempting to hypothesize that the increased gene expression of *TRAIL* in dogs affected by DDD observed in our study is a potential cause for apoptosis, suggesting an involvement of the TRAIL/DR4/DR5 axis and activation of the downstream caspases system. The collective data presented in the literature supports the theory that pro‐inflammatory mediators have an important impact on IVD cell apoptosis and consecutive IVD degeneration.^70^ However, caspases are in a downstream position and their activation is easily regulated by a variety of pro‐producing and pro‐inhibitory signals.^70^ Further analyses certainly are necessary to evaluate the importance of the upregulation of TRAIL expression in distinct IVD regions within the apoptotic process, where the TRAIL/DR4/DR5 axis and the downstream activation of both initiator and activator caspases in IVD cells of dogs suffering from DDD are investigated in depth.^75^

### 4.3 Degeneration of the LF and IVD involves an upregulation of NGF on a protein level

The present study is the first to report increased protein expression of NGF in multiple spinal tissues in a large animal model suffering from naturally occurring DDD and is in line with observations in experimentally induced IVD degeneration in dogs.^76^, ^77^ To our knowledge, the present study is the first to report NGF protein expression in the degenerated LF. The increased NGF protein expression found may highlight that degeneration of the LF involves similar neurotrophic processes as IVD degeneration.

NGF is a known neurotrophin and TNFSF ligand^78^ and has been associated with the development of discogenic pain in humans.^28^, ^29^ It may be hypothesized that, similar to humans, the overexpression of NGF protein in the degenerated canine LF and IVD is a significant contributor to the development of discogenic pain and/or back pain in dogs. In contrast to the protein expression results, NGF gene expression was significantly lower in degenerated IVD tissue compared to healthy controls. This underlines the importance of protein validation after performing qPCR experiments for screening purposes. The discrepancy between RNA and protein expression may be the result of post‐transcriptional and post‐translational mechanisms for controlling protein turnover.^79^ Moreover, specifically, the expression of NGF in the process of IVD degeneration is known to be involved in a complex regulatory network, and pain associated with degenerative IVDs is a function of interacting inflammatory molecules within the IVD, but also retrograde transfer of pain modulators.^80^ The cells of the IVD are known to be both a source and a target for neurotrophins, especially for NGF and brain‐derived neurotrophic factor (BDNF). In contrast to a lack of expression of neutrophins in the healthy IVD, both neurotrophis and neutrophin receptors are expressed at increased levels in painful IVDs.^28^, ^80^, ^81^, ^82^, ^83^ Neutrophins produced in the IVD can be retrogradely transported to cell bodies of sensory neurons where they potentiate nerve growth and expression of specific neurotransmitters, such as substance P or calcitonin gene‐related peptide. Furthermore, neurotrophins are known to induce the expression of pain‐associated cation channels in the dorsal root ganglion. Depolarization of these ion channels is likely to promote discogenic and radicular pain and to reinforce the cytokine‐mediated degenerative cascade.^62^ In turn, neurons of the dorsal root ganglia that innervate the IVD also produce neurotrophins, which can be transported anterogradely into the IVD and interact with neurotrophin receptors expressed in the cells of the AF and nucleus pulposus.^80^ Finally, the expression of both neutrophins and neutrophin receptors in the IVD cells suggests, in addition to the neurotrophic function, an autocrine or paracrine role for these molecules in regulating the biology of the IVD.^80^

### 4.4 RANKL expression is significantly upregulated in degeneration of the IVD, but not of the LF

Historically, RANKL is mainly known to regulate bone remodeling through the RANKL/RANK/osteoprotegerin (OPG) system.^84^ RANKL has even been proposed as a promising target for therapeutic interventions in metabolic bone disease.^85^ More recently, the role of RANKL has also been assessed in other tissues, including the IVD.^86^, ^87^ In accordance with the findings of the present study, increased protein expression of RANKL has been shown in advanced stages of IVD degeneration in people.^87^ Moreover, stimulation of cultured human IVD cells by rhRANKL resulted in a significant upregulation of the catabolic factors MMP‐3 and MMP‐13.^86^ Although RANKL is a transmembrane protein, the location of RANKL immunopositivity observed in the present study was membranous and cytoplasmatic, which can be the result of different RANKL isoforms.^84^, ^88^ RANKL is known to bind to RANK and to activate cytoplasmic adaptor proteins, such as tumor necrosis factor receptor‐associated factor 6. These events activate downstream signaling pathways involving NF‐κB and the MAPK family.^89^ Hence, the intracellular immunopositivity of RANKL observed in the present study may reflect an internalization and activation of downstream signaling pathways. Taken together, the accepted role of RANKL in bone remodeling, the known stimulation of catabolic factors by RANKL, and the high protein expression of RANKL in advanced stages of degeneration especially in cells surrounding structural defects, our findings may highlight an essential role of RANKL in IVD matrix remodeling and degradation. However, further mechanistic studies are needed that focus on the biological and pathological importance of RANKL in the IVD as well as on the contribution of surface and integrated RANKL in the process of DDD.

### 4.5 Anatomical origin of back pain

One major clinical question that emerges from our findings is whether the TNFSF regulations identified in the present study can be linked to clinical pain. Although the dogs used in this study were diagnosed with DLSS and associated pain, the exact anatomical origin of the pain is difficult to localize, especially in animal patients. DLSS involves degeneration of several spinal tissues, including the IVD, LF, but also the facet joints and vertebral endplates. Degenerative changes of the vertebral endplates, so‐called MCs, have been proposed as a cause for low back pain in humans.^90^ More recently, it was shown that DDD in dogs also involves MCs of the vertebral endplates, especially in the lumbosacral junction,^22^ where the presence of lumbosacral MCs was positively associated with age and disc herniation. In our study population, MCs of L7‐S1 were detected in 1/22 (4.5%) of control dogs and 11/18 (61.1%) of dogs of the DDD group (Table 1). The majority of these MCs were type 3 (10/12; 83.3%). These findings are in accordance with Beukers et al.,^22^ showing a positive association between the presence of MCs and age, Pfirrmann Grade, and disc protrusion, and a high prevalence of type III MCs in dogs with degenerative lumbosacral disease. Therefore, in approximately half of the DDD cases of our study, vertebral endplate changes may have been associated with back pain. Retrospective analysis of the qPCR data revealed no significant differences in gene expression of any of the TNFSF members evaluated between samples with and without MCs (data supplied as Table S2). Hence, a clear association between MCs and variations in inflammatory profiles could not be identified based on our data. Moreover, a large proportion of the dogs of the DDD group investigated in our study did not show any MCs (48.9%), indicating that degeneration of other structures, such as the IVD and LF, was likely associated with back pain in these cases. In addition, although MCs appear to be a common phenomenon in dogs with DDD, the reported histological changes associated with these MCs seem to differ from the changes observed in humans.

All in all, although a clear regulation of various inflammatory mediators was identified in the present study, it remains unclear if these mediators can be directly linked to the development of pain observed in these patients. Future studies focusing on the collection of a large number of clinically relevant samples, combined with intensive stratification of MRI to identify pathologic risk factors such as MCs, and linking them to back pain will be necessary.

### 4.6 Study limitations

This study had several limitations. First, a relatively large degree of variation in gene expression was found for both the LF and IVD between donors (Tables 4 and 5). This high degree of variation may have resulted in the absence of significant gene expression regulations for the LF in this study. The high degree of gene expression variation appears to be common in experiments using tissues from human and dog patients^58^, ^91^ and may be explained by several factors. First, a relatively high heterogenicity within the different testing groups and the relatively small number of patients may have caused significant variations in gene expression between donors/testing groups. This heterogenicity can be explained by different factors inherent to the clinical character of the study, including breed‐specific differences and differences in the biological stage of the disease. Second, while working with LF and IVD tissues, we encountered the inherent challenge of low RNA yields, particularly notable in the IVD samples from the DDD group. To address this challenge, we employed a cDNA amplification step that enabled the assessment of multiple targets for a more comprehensive expression profile using limited starting material. It is important to acknowledge that the nature of low yields and small sample volumes can introduce some level of variability and potential error in our experimental procedures. Third, it is reasonable to assume that the inherent instability of mRNA molecules encoding inflammatory mediators could further amplify the observed gene expression fluctuations.^92^

The high level of variation of the gene expression results presented here calls for a cautious interpretation of the gene expression data. Nevertheless, the gene expression variation may also mirror the complexity of the inflammatory processes occurring in vivo: although patient material is inherently varied in contrast to in vitro material, it is the closest representation of disease processes in clinical patients.

### 4.7 Conclusion

Degeneration of the LF and IVD involves a significant regulation of TNFSF members with clear differences between the LF and IVD. DDD involved an upregulation of *CD40LG*, *CD70*, *TNFSF10*, and *RANKL* in the IVD and an upregulation of NGF protein expression in both the LF and IVD. It may be hypothesized that the significant upregulation of these TNFSF members in the IVD results in a modulation of downstream signaling pathways such as NFkB, matrix remodeling/degradation, and sensory innervation of the disc. The TNFSF members may provide interesting targets for future studies directed at regulating the inflammatory/catabolic responses within the degenerating LF and IVD and developing regenerative treatment strategies for patients affected by DDD.

## CONFLICT OF INTEREST STATEMENT

The authors declare no conflicts of interest.

## Supporting information


**FIGURE S1.** Expression stability (*M*‐values) of the individual reference genes and pairwise variation (*V*‐value) for the ligamentum flavum obtained using geNorm reference gene analysis.

**FIGURE S2.** Expression stability (*M*‐value) of the individual reference genes and pairwise variation (*V*‐value) for the intervertebral disc obtained using geNorm reference gene analysis.

**SUPPLEMENTARY FILE 1:** RNA Isolation for ligamentum flavum and intervertebral disc tissue.

**SUPPLEMENTARY FILE 2.** Average expression stability of reference genes for the ligamentum flavum and the intervertebral disc.

**SUPPLEMENTARY FILE 3.** Western blot analysis.

**SUPPLEMENTARY FILE 4.** Immunohistochemistry of RANKL (TNFSF11).

**TABLE S1.** *p* Values for comparisons between control and degenerated (DDD) ligamentum flavum and between different degeneration stages (Thompson grades I–V) for the nucleus pulposus (NP), dorsal annulus fibrosus (AF), and ventral AF. Also, *p* Values for the comparison between non‐chondrodystrophic (NCD) and chondrodystrophic (CD) dogs for the three evaluated disc regions are displayed.

**TABLE S2.** Median (range) for dCT values for IVD samples with (MC+) and without (MC−) Modic changes of the vertebral endplates. Because of the small sample size and unequal distribution between groups, the data were considered non‐normally distributed and median (range) for the dCT values have been displayed. *p* Values for the comparison between MC+ and MC− groups (Mann–Whitney *U* test) have been displayed for each gene target, showing no significant differences between the MC+ and MC− groups. MC, Modic changes; NE, No expression.

Appendix I
