# Supplementary figures and images for: Targeted screening of inflammatory mediators in spontaneous degenerative disc disease in dogs reveals an upregulation of the tumor necrosis superfamily
Source: JOR Spine. 2023 Nov 23;7(1):e1292. doi: 10.1002/jsp2.1292 (PMC10782068; doi:10.1002/jsp2.1292)

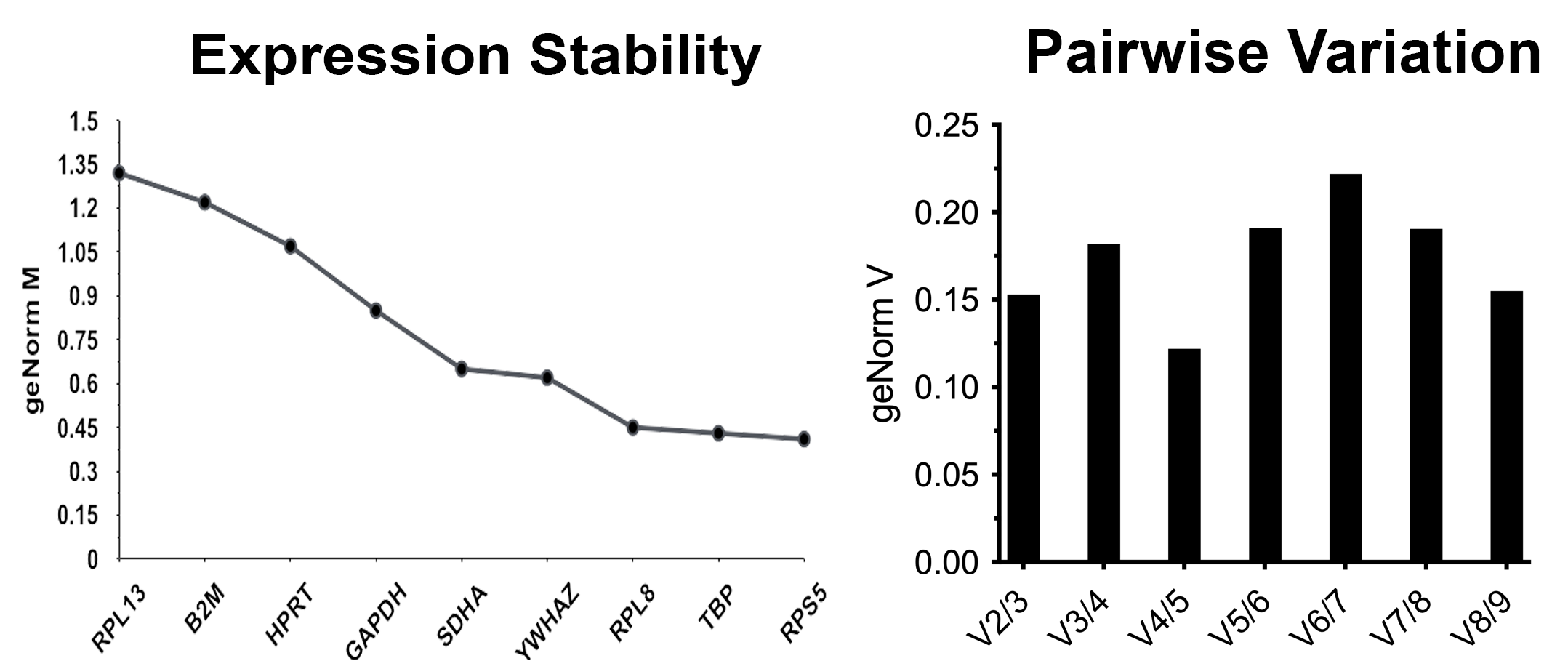

Supplement: Supplementary file 1 — FIGURE S1. Expression stability (M‐values) of the individual reference genes and pairwise variation (V‐value) for the ligamentum flavum obtained using geNorm reference gene analysis. [file JSP2-7-e1292-s006.tif]

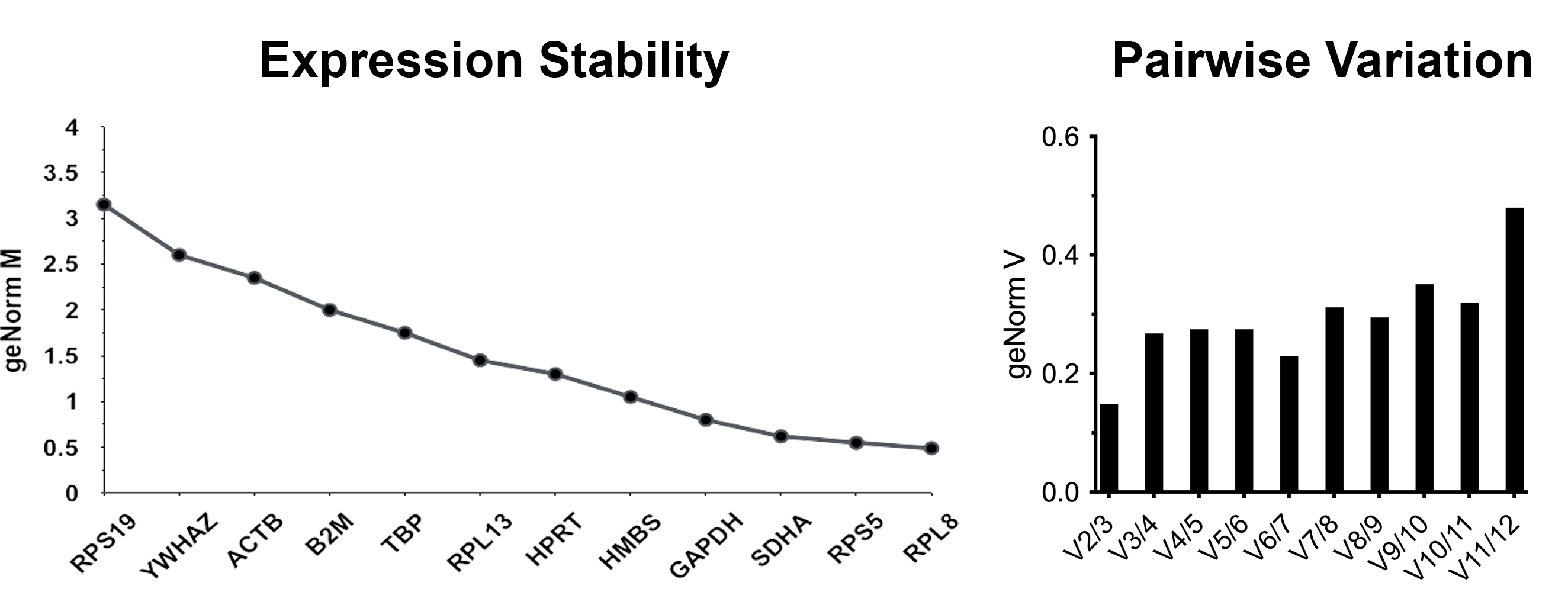

Supplement: Supplementary file 2 — FIGURE S2. Expression stability (M‐value) of the individual reference genes and pairwise variation (V‐value) for the intervertebral disc obtained using geNorm reference gene analysis. [file JSP2-7-e1292-s008.tif]
