# Supplementary material for: Targeted screening of inflammatory mediators in spontaneous degenerative disc disease in dogs reveals an upregulation of the tumor necrosis superfamily
Source: JOR Spine. 2023 Nov 23;7(1):e1292. doi: 10.1002/jsp2.1292 (PMC10782068; doi:10.1002/jsp2.1292)
Supplement: Supplementary file 3 — SUPPLEMENTARY FILE 1: RNA Isolation for ligamentum flavum and intervertebral disc tissue. [file JSP2-7-e1292-s004.doc]

**Supplementary File 1: RNA Isolation for ligamentum flavum and intervertebral disc tissue**

For RNA extraction, fresh frozen tissue samples stored at -80° C were transferred into a precooled mortar filled with liquid nitrogen and manually grinded under RNAse-free conditions. The frozen tissue powder was transferred into 1 ml of Trizol solution (ThermoFisher, 15596-026). Samples were homogenized twice for 30 seconds using a Polytron (Polytron PT 2500E, Kinematica, Luzern, Switzerland). After homogenization the samples were incubated for 30 minutes at room temperature on an orbital shaker and centrifuged at 12000 *g* for 10 minutes at 4° C. The supernatant was transferred in a new tube, and 200 µl chloroform diluted in 1 ml Trizol was added, shaken for 15 seconds on a Vortex (Vortex-Genie 2, Scientific Industries Inc, New York, USA), and incubated another 15 minutes at room temperature. Afterwards, the mixture was centrifuged with 12000 *g* for 15 minutes at 4° C. Then, 500 µl per 1 ml Trizol of the aqueous phase was transferred into a new tube, and the same volume of 70 % ethanol was added and mixed.

The NuceloSpin RNA extraction kit (NucleoSpin RNA Kit 74099.50, Marcherey-Nagel, Oensingen, Switzerland) was used for RNA extraction. The mixture was transferred up to 700 µl of the sample, including any precipitate that may have formed, to a NucleoSpin column placed in a 2 ml collection tube, and centrifuged for 30 seconds with 8000 g at 22° C. If the sample volume exceeded 700 μl, multiple aliquots were centrifuged in the same spin column. The flow-through was discarded after each centrifugation. The spin column membrane was placed in a new collection tube and 350 µl of MDB buffer (NucleoSpin RNA Kit 74099.50, Marcherey-Nagel) was added to the spin column and centrifuged for 1 minute with 11000 *g* at 22° C to wash the spin column membrane. Subsequently, the flow-through was discarded.

For DNase treatment, 10 μl reconstituted rDNase solution was gently mixed with 90 μl reaction buffer, and 95 µl rDNase incubation mix was added directly on to the spin column membrane and incubated at room temperature for 15 minutes. To inactivate the DNase, 200 μl RAW2 buffer (NucleoSpin RNA Kit 74099.50, Marcherey-Nagel) was added to the spin column and centrifuged for 30 seconds with 11000 *g* at 22° C. The flow-through was discarded, and the column was placed into a new collection tube.

Spin columns were washed twice using RA3 buffer (NucleoSpin RNA Kit 74099.50, Marcherey-Nagel), first using 600 µl RA3 buffer centrifuged with 11000 *g* for 30 seconds and a second time using 250 µl centrifuged with 11000 *g* at 22 ° C for 2 minutes. The flow-through was again discarded.

The spin column membranes were dried with 11000 *g* at 22° C for 30 seconds. Purified RNA-containing NucleoSpin column membranes were placed in a new 1.5 ml collection tube and 40 µl RNase -free water was added directly to the membrane and incubated for 5 minutes at room temperature. Membranes were subsequently centrifuged for 1 minute with 11000 *g* to elute the RNA from the membrane. The quantity and quality of the isolated RNA was determined using NanoDrop 1000 (NanoDrop 1000, Thermo Fisher Scientific, Basel, Switzerland).

The isolated RNA was stored on ice or for long-term storage at -80° C.
