# Supplementary material for: Targeted screening of inflammatory mediators in spontaneous degenerative disc disease in dogs reveals an upregulation of the tumor necrosis superfamily
Source: JOR Spine. 2023 Nov 23;7(1):e1292. doi: 10.1002/jsp2.1292 (PMC10782068; doi:10.1002/jsp2.1292)
Supplement: Supplementary file 4 — SUPPLEMENTARY FILE 2. Average expression stability of reference genes for the ligamentum flavum and the intervertebral disc. [file JSP2-7-e1292-s005.docx]

**Supplementary file 2**

**Average expression stability of reference genes for the ligamentum flavum and the intervertebral disc**

Optimal reference genes were determined separately for the ligamentum flavum (LF) and the intervertebral disc (IVD). Five samples per tissue for both the control and DDD group were included. Twelve commonly used housekeeping genes *beta actin* (*ACTB), beta 2 microglobulin (B2M), glyceraldehyde 3-phosphate dehydrogenase (GAPDH), hydroxymethylbilane synthase (HMBS), hypoxanthine-guanine phosphoribosyltransferase (HPRT), 60S ribosomal protein L8 (RPL8), 60S ribosomal protein L13 (RPL13), 40S ribosomal protein S5 (RPS5), 40S ribosomal protein S19 (RPS19), succinate dehydrogenase complex subunit a (SDHA), TATA-box binding protein (TBP), tyrosine 3-monooxygenase/tryptophan 5-monooxygease activation protein zeta (YWHAZ)* were included and tested for expression stability in LF and IVD. The expression stability (M-value <0.5 is desirable) and the optimal number (V-value of <0.15 is desirable) were calculated using geNorm analysis.

**Results**

For the LF, *ACTB*, *RPS19*, *HMBS* were excluded for geNorm analysis due to inconsistent expression. The inclusion of four housekeeping genes provided the best stability for normalization (Supplementary Figure 1). These reference genes included *RPS5, TBP, RPL8* and *YWHAZ*.

For the IVD, inclusion of three housekeeping genes provided the best stability for normalization (Supplementary Figure 2). These reference genes included *SDHA, RPL8, RPS5*.
