# Supplementary material for: Targeted screening of inflammatory mediators in spontaneous degenerative disc disease in dogs reveals an upregulation of the tumor necrosis superfamily
Source: JOR Spine. 2023 Nov 23;7(1):e1292. doi: 10.1002/jsp2.1292 (PMC10782068; doi:10.1002/jsp2.1292)
Supplement: Supplementary file 5 — SUPPLEMENTARY FILE 3. Western blot analysis. [file JSP2-7-e1292-s009.docx]

**Western Blot Analysis**

For protein validation of the gene expression results, Western Blot analysis for the protein target Nerve Growth Factor (NGF) and Collagen I was performed using four healthy and four degenerated ligamentum flavum and intervertebral disc samples which were, if possible, complementary to the gene expression samples (Table 1). Tissues were homogenized using RIPA buffer, EDTA and proteinase inhibitor (Halt Protease Inhibitor, Thermo Fischer Scientific, Wilmington, USA). Homogenates were centrifuged for 20 minutes at 12.000 x g at 4^o^ C and the total protein concentration of the supernatant was determined using a Bradford Protein Assay (Bio-Rad Protein Assay Dye Reagent Concentrate, Bio-Rad Laboratories, Cressier, Switzerland). NGF and Collagen I were detected using canine specific antibodies according to manufacturer’s protocol. Polyarcrylamid SDS TGX Gels 4-20% (Biorad) were loaded with a total amount of 25mg of protein per well, which were then separated using electrophoresis at a voltage of 200V for approximately 25 minutes. Additionally, a positive control and a ladder were included on each gel. The gels were subsequently activated with Stain-Free program using the ChemiDoc Touch (Biorad). The Stain-Free technology utilizes a special polyacrylamide gel chemistry, which allows to make proteins fluorescent directly in the gel with photoactivation. Thereby, visualization of proteins is possible at any timepoint during electrophoresis and blotting. The separated proteins were blotted onto nitrocellulose membranes using Transturbo Blot (Biorad, midi 7 Minutes). The membranes were washed three times for 10 minutes with Tris-buffered saline + Tween 20 (TBS-T) solution. After washing, non-specific bindings were blocked with a TBS-T + 5% milk solution (RapiLait Magermilchpulver, Migros, Switzerland) at room temperature for two hours, after which the membranes were taken through another washing step with TBS-T for three times 10 minutes. The washed membranes were incubated with the primary antibody diluted in TBS-T + 1% milk over night with gentle agitation in a cold room (4°C). Another washing step was performed. The secondary antibody was added to the membrane, diluted 1:2500 in TBS-T + 1% milk, and incubated for 1 hour at room temperature. The membranes were washed again. Antibody detection was performed with ECL Western Bright (Biorad) using the ChemiDoc Touch (Biorad). Protein expression was quantified using a Chemidoc Touch System and ImageLab software (Bio-Rad). For each lane, total protein concentration was determined per sample using Stain Free Technology. The band signal for the protein of interest of each lane was subsequently normalized for the total sample protein content to determine the relative expression of each protein of interest within each sample. Normalized protein expression was used as a quantitative measure and used for statistical analysis.
