# Supplementary material for: Targeted screening of inflammatory mediators in spontaneous degenerative disc disease in dogs reveals an upregulation of the tumor necrosis superfamily
Source: JOR Spine. 2023 Nov 23;7(1):e1292. doi: 10.1002/jsp2.1292 (PMC10782068; doi:10.1002/jsp2.1292)
Supplement: Supplementary file 6 — SUPPLEMENTARY FILE 4. Immunohistochemistry of RANKL (TNFSF11). [file JSP2-7-e1292-s002.docx]

**Immunohistochemistry of RANKL (TNFSF11)**

RANKL protein expression was evaluated in ligamentum flavum and intervertebral disc tissue. After deparaffinization, sections were washed with phosphate-buffered saline (PBS) solution and permeabilized using 0.2% triton X 100 solution (15 minutes), followed by washing with PBS and blocking of endogenous peroxidase activity (3% H_2_O_2_ + 0.2% NaN_3_ solution; 10 minutes). Nonspecific background staining was minimized by pre-incubation with protein blocking solution (Dako X0909, Baar, Switzerland) for 10 minutes. Sections were incubated overnight at 4^0^ C with a primary anti-RANKL polyclonal antibody (Abcam, ab216484, 1:200 in PBS). After sections were washed in PBS buffer, sections were incubated with a secondary antibody (Envision Dual Link System-HRP, Dako K4061) and RANKL staining was visualized with DAB substrate (Dako, K3468). Sections were counterstained with hematoxylin (Hematoxylin QS, Vector Laboratories Inc.) and mounted using Aquatex solution. Positive controls included canine lymph node tissue. In negative control sections, the primary antibody was omitted.
