# Supplementary material for: Targeted screening of inflammatory mediators in spontaneous degenerative disc disease in dogs reveals an upregulation of the tumor necrosis superfamily
Source: JOR Spine. 2023 Nov 23;7(1):e1292. doi: 10.1002/jsp2.1292 (PMC10782068; doi:10.1002/jsp2.1292)
Supplement: Supplementary file 7 — TABLE S1. p Values for comparisons between control and degenerated (DDD) ligamentum flavum and between different degeneration stages (Thompson grades I–V) for the nucleus pulposus (NP), dorsal annulus fibrosus (AF), and ventral AF. Also, p Values for the comparison between non‐chondrodystrophic (NCD) and chondrodystrophic (CD) dogs for the three evaluated disc regions are displayed. [file JSP2-7-e1292-s001.docx]

| **Ligamentum flavum** |  |  |  |
| --- | --- | --- | --- |
| Control vs. DDD | 0.1306 |  |  |
|  |  |  |  |
| **Intervertebral disc** | *NP* | *Dorsal AF* | *Ventral AF* |
| Thompson 1 vs. 2 | 0.0678 | 0.5613 | 0.8341 |
| Thompson 1 vs. 3 | 0.4485 | 0.2184 | 0.9110 |
| Thompson 1 vs. 4 | **0.0004** | 0.1176 | 0.9565 |
| Thompson 1 vs. 5 | **0.0001** | **0.0050** | **0.0001** |
| Thompson 2 vs. 3 | 0.2937 | 0.4907 | 0.9583 |
| Thompson 2 vs. 4 | **0.0185** | 0.2789 | 0.8663 |
| Thompson 2 vs. 5 | **0.0001** | **0.0168** | **0.0001** |
| Thompson 3 vs. 4 | **0.0028** | 0.6559 | 0.9342 |
| Thompson 3 vs. 5 | **0.0001** | **0.0331** | **0.0001** |
| Thompson 4 vs. 5 | **0.0001** | **0.0194** | **0.0001** |
|  |  |  |  |
| NCD vs. CD | 0.6457 | 0.953 | 0.7149 |

**Supplementary Table 1:** P-values for comparisons between control and degenerated (DDD) ligamentum flavum and between different degeneration stages (Thompson Grades I-V) for the nucleus pulposus (NP), dorsal annulus fibrosus (AF) and ventral AF. Also, P-values for the comparison between non-chondrodystrophic (NCD) and chondrodystrophic (CD) dogs for the three evaluated disc regions are displayed.
