# Supplementary material for: Targeted screening of inflammatory mediators in spontaneous degenerative disc disease in dogs reveals an upregulation of the tumor necrosis superfamily
Source: JOR Spine. 2023 Nov 23;7(1):e1292. doi: 10.1002/jsp2.1292 (PMC10782068; doi:10.1002/jsp2.1292)
Supplement: Supplementary file 8 — TABLE S2. Median (range) for dCT values for IVD samples with (MC+) and without (MC−) Modic changes of the vertebral endplates. Because of the small sample size and unequal distribution between groups, the data were considered non‐normally distributed and median (range) for the dCT values have been displayed. p Values for the comparison between MC+ and MC− groups (Mann–Whitney U test) have been displayed for each gene target, showing no significant differences between the MC+ and MC− groups. MC, Modic changes; NE, No expression. [file JSP2-7-e1292-s003.docx]

| Intervertebral Disc | | | |
| --- | --- | --- | --- |
| Target | Diseased MC + (n=6) | Diseased MC- (n=2) | P-value |
| *NGF* | 7.01 (4.98 – 8.20) | 7.31 (6.87 – 7.75) | 0.952 |
| *NGFR* | 9.18 (6.55 – 10.71) | 9.99 (9.45 – 10.54) | 0.857 |
| *TNF-a* | 9.55 (9.34 – 11.05) | 10.35 (9.48 –11.22) | 0.857 |
| *LTA* | NE | NE |  |
| *LTB* | NE | NE |  |
| *CD40LG* | 11.68 (11.43 – 18.15) | 11.41 (9.67 – 13.14) | 0.857 |
| *FasL* | 13.91 (10.7 – 20.50) | 12.75 (11.99 – 13.52) | 0.857 |
| *CD70* | 8.05 (7.18 – 19.11) | 10.17 (8.22 – 12.12) | 0.571 |
| *TNFSF10* | 2.17 (1.80 – 2.86) | 2.26 (1.99 – 2.53) | 1.000 |
| *TNFSF11* | 4.68 (3.61 – 5.47) | 6.48 (4.84 – 8.13) | 0.381 |
| *TNFSF13B* | 4.26 (3.44 – 4.86) | 2.99 (2.58 – 3.41) | 0.095 |
| *TNFSF14* | 14.24 (13.67 – 19.11) | 16.64 (16.07 – 17.21) | 0.857 |

**Supporting Table 2:** Median (range) for dCT values for IVD samples with (MC+) and without (MC-) Modic changes of the vertebral endplates. Because of the small sample size and unequal distribution between groups, the data was considered non-normally distributed and median (range) for the dCT values have been displayed. P values for the comparison between MC+ and MC- groups (Mann-Whitney-U test) have been displayed for each gene target, showing no significant differences between the MC+ and MC- groups. MC = Modic changes; NE = No expression.
