# Supplementary material for: Targeted screening of inflammatory mediators in spontaneous degenerative disc disease in dogs reveals an upregulation of the tumor necrosis superfamily
Source: JOR Spine. 2023 Nov 23;7(1):e1292. doi: 10.1002/jsp2.1292 (PMC10782068; doi:10.1002/jsp2.1292)
Supplement: Supplementary file 9 — Appendix I [file JSP2-7-e1292-s007.pdf]

University of Zurich[UZH]

Animal Welfare and 3R

University of Zurich
Office for Animal Welfare and 3R
Winterthurerstrasse 190
CH-8057 Zurich

http://www.tierschutz.uzh.ch/index_en.html

UZH, Dept Animal Welfare and 3R, Wintherthurerstr. 190,CH-8057 Zurich

Dr. Michaela Thallmair
Head Office for Animal Welfare and 3R
Animal Welfare Officer

+41 44 635 82 92
michaela.thallmair@uzh.ch

Journal of Orthopedic Research: Spine
Editorial Team:
Dr. Mauro Alini, Dr. Robert Mauck & Dr. Daisuke Sakai

Zurich, May 15th 2023

Dear Editorial Team

Switzerland has very strict regulations for animal experiments. Article 3 of the Animal Welfare Act (Tierschutzgesetz) defines what an animal experiment is:

Art 3 c. animal experiment: Any measure in which a live animal is used with the aim of
1. testing a scientific assumption,
2. observing the effect of a particular measure in the animal,
3. testing a substance,
4. obtaining or testing cells, organs or bodily fluids, except when this is in the context of agricultural production, diagnostic or curative operations on the animal or for determining the health status of animal populations,
5. obtaining or replicating organisms alien to the species in question,
6. teaching or training.

All of these procedures need an ethical review by the cantonal animal experimentation commission and – if deemed appropriate and necessary – will get a license by the competent authority (veterinary office).

If material is taken from animals that have been euthanized or killed for other reasons, e.g. medical reasons (animal hospital), i.e. the reason for the animals' death has no relation to and was not influenced by the experimental research aim, no animal experimentation license is needed.

In the manuscript "Upregulation of Tumor Necrosis Superfamily Members in a Dog Model of Spontaneous Degenerative Disc Disease" Dr. Luc Smolders used material from cadavers after dogs were euthanized based on medical necessity. Since Dr. Smolders confirmed that the collection of these and the euthanasia of the dogs was not related my office confirmed that no animal license was needed, but written consent by the owners.

Please do not hesitate to get back to me in case of questions.

Best regards

Michaela Thallmair

Dr. Michaela Thallmair, Head Office for Animal Welfare and 3R
